# The Role of Cholesterol in M2 Clustering and Viral Budding Explained

**DOI:** 10.1021/acs.jctc.4c01026

**Published:** 2024-11-04

**Authors:** Dimitrios Kolokouris, Iris E. Kalenderoglou, Anna L. Duncan, Robin A. Corey, Mark S. P. Sansom, Antonios Kolocouris

**Affiliations:** 1 Laboratory of Medicinal Chemistry, Section of Pharmaceutical Chemistry, Department of Pharmacy, National and Kapodistrian University of Athens, Panepistimiopolis Zografou, Athens 15771, Greece; 2 Department of Biochemistry, University of Oxford, Oxford OX1 3QU, U.K.; 3 School of Physiology, Pharmacology and Neuroscience, University of Bristol, Bristol BS8 1TD, U.K.

## Abstract

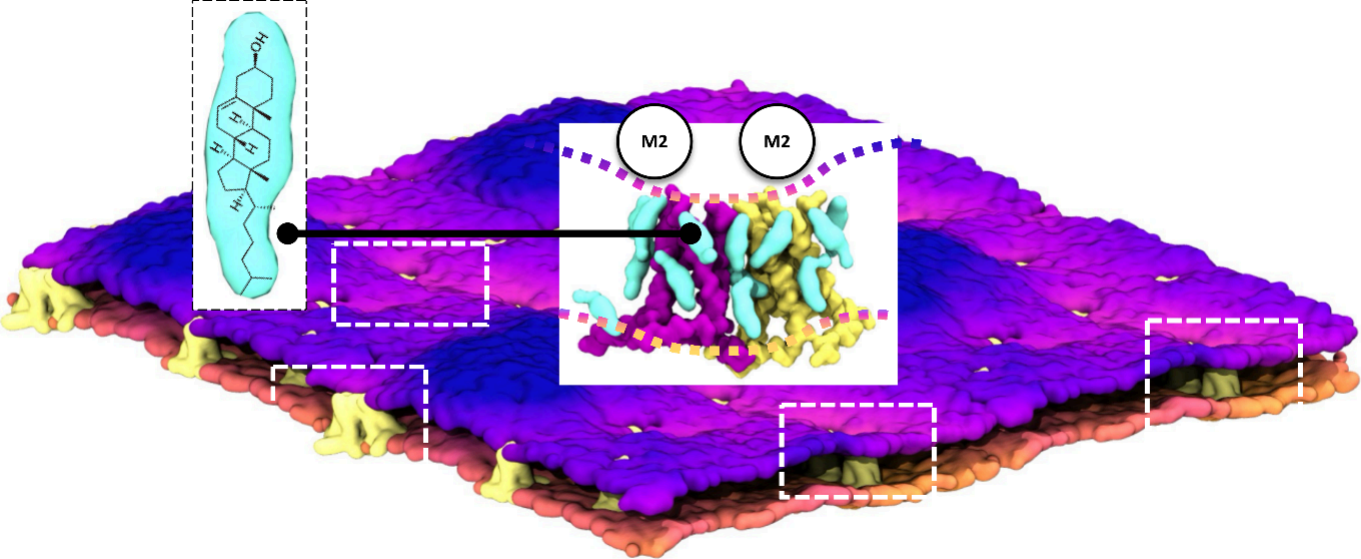

The influenza A M2 homotetrameric channel consists of four transmembrane (TM) and four amphipathic helices (AHs). This viral proton channel is suggested to form clusters in the catenoid budding neck areas in raft-like domains of the plasma membrane, resulting in cell membrane scission and viral release. The channel clustering environment is rich in cholesterol. Previous experiments have shown that cholesterol significantly contributes to lipid bilayer undulations in viral buds. However, a clear explanation of membrane curvature from the distribution of cholesterol around the M2TM-AH clusters is lacking. Using coarse-grained molecular dynamics simulations of M2TM-AH in bilayers, we observed that M2 channels form specific, C2-symmetric, clusters with conical shapes driven by the attraction of their AHs. We showed that cholesterol stabilized the formation of M2 channel clusters by filling and bridging the conical gap between M2 channels at specific sites in the N-termini of adjacent channels or via the C-terminal region of TM and AHs, with the latter sites displaying a longer interaction time and higher stability. The potential of mean force calculations showed that when cholesterols occupy the identified interfacial binding sites between two M2 channels, the dimer is stabilized by 11 kJ/mol. This translates to the cholesterol-bound dimer being populated by almost 2 orders of magnitude compared to a dimer lacking cholesterol. We demonstrated that the cholesterol-bridged M2 channels can exert a lateral force on the surrounding membrane to induce the necessary negative Gaussian curvature profile, which permits spontaneous scission of the catenoid membrane neck and leads to viral buds and scission.

## Introduction

The influenza A M2 proton channel is a canonical viroporin^1−3^ and is one of 17 proteins encoded by the influenza A viral RNA.^4,5^ Four M2 peptides (97 amino acids each) assemble with 4-fold symmetry into the pH-dependent proton conducting M2 channel, whose transmembrane (TM) domain forms the pore of the proton channel.^6−8^ The M2 peptide is a single-pass transmembrane protein with an unstructured N-terminus, a single-pass TM α-helix (M2TM; residues 22–46),^4,6,9,10^ followed by an amphipathic α-helix (AH; residues 47–62)^11^ and a highly dynamic C-terminus (residues 62–97).^12,13^ The M2 channel is blocked by adamantyl amine drugs, which bind inside the proton conducting M2TM pore.^10,14−18^ However, the extended M2TM-AH, consisting of both the TM and AH domains, forms the conductance domain of the channel (residues 22–62) having specific, amantadine-sensitive proton transport activity indistinguishable from that of full-length M2 (M2FL).^19^ During endocytosis of viral particles, the M2 channel is activated by low pH, which causes the imidazole side chains of the His37 tetrad in the H^+^-pore to become protonated. The electrostatic repulsion between the His37-H^+^ side chains^20^ drives the M2 channel to adopt an open conformation, resulting in proton influx.^21,22^ The acidification of the viral core leads to unpacking of the viral genome and pathogenesis.^23^ M2TM-AH adopts a wedge- or conical-shaped structure,^11^ embedding the larger lower half of the cone in the inner (cytosolic) leaflet of the biological membrane. This wedge, in turn, generates the required saddle-shaped (negative Gaussian) curvature^24−31^ required for membrane scission and viral budding.^32−37^ Thus, the M2TM-AH segment is the functionally important unit of M2, performing both the H^+^-channel and membrane-curving roles.

Indeed, recent studies using fluorescence and electron microscopy also found M2 channels to concentrate at the neck of the budding virus in the host’s plasma membrane,^32−37^ at the boundary between the raft-like and nonraft areas in the membrane, corresponding to L_d_ and L_o_ phases. Depending on lipid composition and M2 concentration, the wedge-like M2TM-AH structure allows the channel to both induce and sense curvature, concentrating copies of M2 channels according to biochemical,^27,30,32,37^ ssNMR,^25,28,29^ CG MD simulations,^24,29^ and other biophysical studies.^25,26,29,31,33,38^ M2TM-AH clustering and its membrane curvature effect is stronger in phosphatidylethanolamine (PE) membranes than in phosphatidylcholine (PC) or phosphatidylglycerol (PG) membranes according to ssNMR studies.^31^ Membrane curvature sensing/generation is realized by protein–protein interactions (PPIs) between AHs^39^ as has been reviewed for a plethora of membrane-bound proteins,^40,41^ including ion channels.^42^ M2 PPIs differ between channel constructs, including bulky hydrophobic TM residues in M2TM clusters. In contrast, M2TM-AH clusters did not involve TM domain residues to the same degree but instead clustered via the AHs, as shown with ssNMR in DOPC/DOPE (DO: 1,2-dioleoyl) lipid bilayers.^28^ At high local M2 concentrations, as clustering proceeds, the membrane curving effect is exacerbated, and influenza A budding begins via the release of the newly constituted virions,^32,33,35−37^ attracting more M2 copies to budding necks. Thus, the M2 channel AHs facilitate curvature and budding,^24−31,43−45^ replacing the need for the endosomal sorting protein complexes required for transport (ESCRT) machinery proteins utilized by other viruses.^32,33,36,37^

Several studies have shown that cholesterol can influence the oligomerization state and energy landscape of, e.g., amyloid-β protein oligomers^46^ or HIV Fusion Protein gp41.^47^ Cholesterol significantly contributes to lipid bilayer undulations in viral buds,^27,48−50^ implying its involvement in the budding process. Similarly, the highly curved neck of an influenza A viral bud is characterized by phase separation involving cholesterol and lipid segregation.^34,51^ ssNMR^25,28,29,31^ and CG MD investigations^24^ have demonstrated that the M2 channel tends to locate itself at the boundary between the raft-like and nonraft areas in the membrane,^52,53^ where the budding virus can enrich itself with cholesterol to build its viral envelope.^27,48−50^ While elevated levels of POPE (PO: 1-palmitoyl-2-oleoyl) lipid suppress the capability of M2TM-AH to induce membrane pits,^49^ cholesterol enhances the ability of M2TM-AH to generate local membrane curvature and pits.^25,27,29,31,49^ Additionally, atomic force microscopy (AFM) and electron paramagnetic resonance (EPR) spectroscopy,^49^ fluorescence emission spectroscopy using FITC-M2TM-AH (FITC: fluorescein isothiocyanate),^54^ electron paramagnetic resonance (EPR), and ^19^F ssNMR^31^ suggested the influence of cholesterol on the position and orientation of M2TM-AH.

M2-cholesterol interactions are recognized to be specific. Results from ssNMR using labeled cholesterols revealed that cholesterol has a binding site on M2 channel constructs in viral and plasma membrane mimetics, including anionic lipids. More specifically, cholesterol binding was detected in (a) M2FL in POPC/POPG/cholesterol bilayers^55^ and (b) M2TM-AH and M2 (22–97) in POPC/POPE/POPS/sphingomyelin (SPH)/cholesterol bilayers (PS: phosphatidylserine).^56,57^ The cholesterol isooctyl tail interacts with the membrane facing Ile39 in M2TM, while the cholesterol polar head lies close to Phe47 at the beginning of AH. Docking predicted that other TM residues, such as Leu43 and Leu46, may accommodate the β-face of the hydrophobic steroid core.^57^ A penta-alanine mutation in AH residues (M2–5Ala: F47A/F48A/I51A/Y52A/F55A)^27^ led to desensitization of M2 conformational dynamics to cholesterol compared to WT, suggesting the importance of the AH domain in cholesterol sensation and conformational regulation. However, the penta-alanine mutation in AH residues was not necessarily budding-defective due to loss of cholesterol binding. These observations agreed with previous results, which showed interaction of cholesterol^27,49^ deep in the bilayer interface with AHs. Overall, it is now believed that the AH domain interacts directly with cholesterol to facilitate bilayer curvature.

However, it remains unclear how cholesterol is directly involved in the clustering of the tetrameric M2 bundles as the mechanism by which the negative curvature neck of the budding virion is formed. CG MD simulations provide a particularly suitable method to explore the interplay between the lipid bilayer composition and the clustering of M2 channels. CG MD simulations have been extensively used to investigate the protein–lipid interactions in membranes with cholesterol,^58,59^ the effect of lipids on the structure and function of membrane peptides and proteins,^40,58,59^ and the membrane curvature and plasticity,^39,60−63^ providing significant insights on membrane dynamics and organization that can be linked directly to experiments.^64−67^ Large-scale CG MD simulations have also been used to investigate protein crowding and its effect on membrane structure and organization.^40,42^

Previous CG MD simulations showed that M2TM-AH formed linear clusters, which generated spontaneous membrane curvature in agreement with experimental findings.^24−30^ However, M2TM-AH clustering studied by CG MD simulations in refs (28) and (24) did not include cholesterol, a major component of the mammalian plasma membrane enriched in raft-like domains and L_o_/L_d_ interfaces.^52,53,68,69^ Here, we used CG MD simulations to show that M2TM-AH embedded in membranes composed of different lipids,^49,70^ with 20% cholesterol or without cholesterol present, forms multimeric assemblies. In line with experiment, we observed that M2 multimers are stabilized and induce membrane curvature in bilayers where cholesterol is present. We showed that cholesterol binds between M2TM-AH protomers in specific interprotein clefts in the top and bottom membrane leaflets with high occupancies. The top leaflet sites are more dynamic and showed high exchange rates, whereas the bottom leaflet sites had slower off-rates, favoring the formation of M2TM-AH multimers. We quantified the effect of cholesterol on M2TM-AH clustering via umbrella sampling (US) potential of mean force (PMF) calculations of the dimerization free energies. Cholesterol-induced M2TM-AH clusters increased the Gaussian curvature of the surrounding membrane, a precursor of the budding process.

## Results

### Simulated Systems

We applied CG MD simulations for systems including 16 copies of M2TM or M2TM-AH channels in lipid bilayers consisting of 5,000 lipid molecules. In order to explore the effect of M2 clustering^24,28,31^ and cholesterol^26,27,29,31^ on membrane bending, we performed CG MD simulations for M2TM channels in DMPC (DM: 1,2-dimyristoyl) and POPC bilayers, the latter without or with 20% cholesterol, for M2TM-AH channels in DMPC, POPC, and POPC/POPS bilayers, the latter two without or with 20% cholesterol, and for lipid-only bilayers. DMPC, POPC and POPC/POPS^30,33,37^ bilayers have been applied as membrane models in experimental^30,33,37^ and simulation studies of M2TM-AH clustering and membrane bending. The POPC/POPS lipids are present in mammalian plasma membranes, which have a negative surface charge in the intracellular leaflet due to the presence of the anionic PS lipids and phosphatidylinositol (PI) lipids, e.g., phosphatidylinositol 4,5-bisphosphate (PIP2),^43^ and have been used as virus mimetic membranes in studies of M2.^56,57^ We also performed CG MD simulations for M2TM-AH in a plasma membrane mimetic model. The stability of the dimers of M2TM-AH channels was tested using atomistic (AA) MD simulations. An overview of our simulations is provided in Table S1.

### M2TM and M2TM-AH Clustering

**Figure 1:**
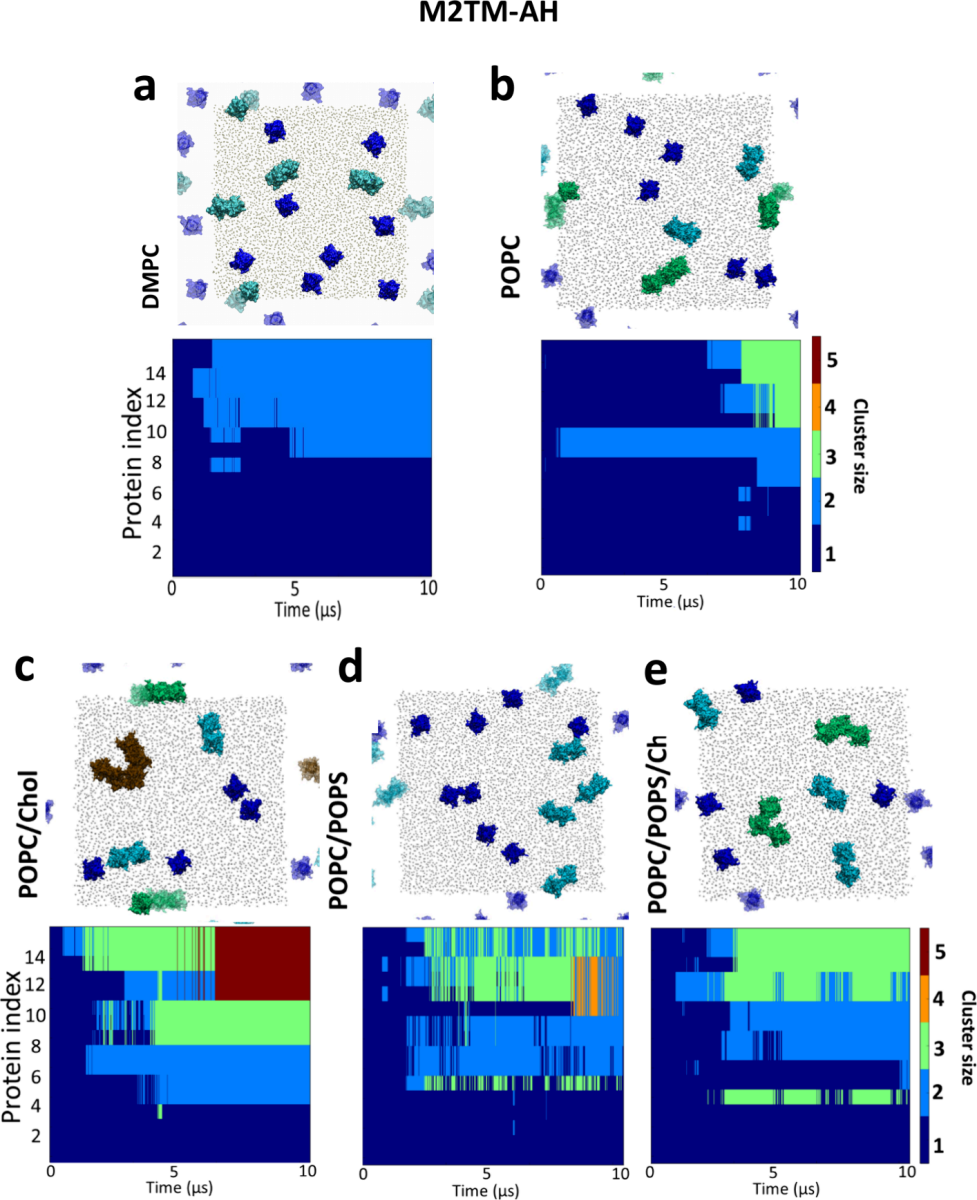
M2TM-AH channel cluster formation over the duration of 10 μs production simulations with the Martini force field.^64,72,73^ The graphs illustrate the time course of M2 channel cluster formation. Sixteen copies of M2 were simulated in bilayers consisting of (a) DMPC; (b) POPC; (c) POPC/cholesterol (4:1); (d) POPC/POPS (4:1); (e) POPC/POPS/cholesterol (3:1:1) (see Table S1 for M2TM clustering). The cluster size is indicated by color (blue for single M2TM-AH protomers, cyan for dimers, green for trimers, orange for tetramers, and brown for pentamers). A representative snapshot at *t* = 10 μs of each system is shown as a top view (*xy* periodicity is shown in transparent).

In all of the membrane systems with 16 copies of M2TM or M2TM-AH tetrameric bundles, we observed that M2 channels clustered, forming dynamic assemblies ranging from dimers to pentamers of the tetrameric channel protein. This analysis aims to predict the kinetics of M2TM-AH cluster formation, starting from the same multiprotein configuration under differing membrane compositions in nonequilibrium conditions. The evolution over time of M2TM-AH clustering in DMPC, POPC, POPC/POPS, POPC/cholesterol, and POPC/POPS/cholesterol is illustrated in Figure 1, and for M2TM in DMPC, POPC, and POPC/cholesterol in Figure S1. Simulation repeats (Table S1) showed similar results. While M2TM formed a maximum cluster size of four channels in DMPC or POPC or POPC/cholesterol (Figure S1), M2TM-AH formed dimers in DMPC (Figure 1a) and POPC/POPS (Figure 1d). In the POPC and POPC/POPS/cholesterol system, M2TM-AH formed dimers to trimers (Figure 1b,e, respectively), and the biggest cluster–a pentamer–was observed in POPC/cholesterol (Figure 1c), suggesting cholesterol increases the rate and order of oligomers.

**Figure 2:**
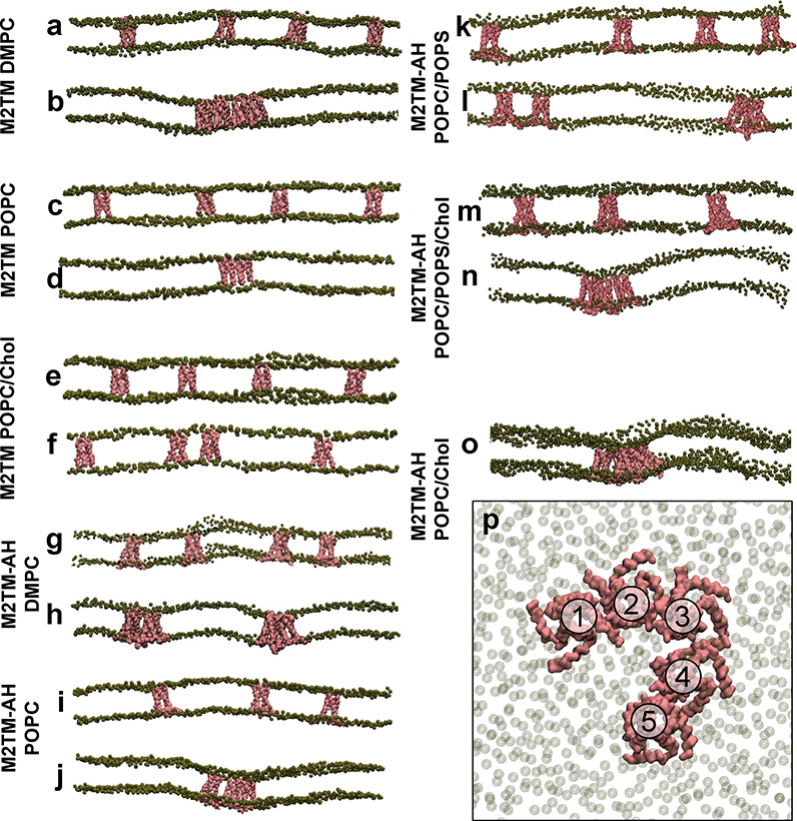
Initial (*t* = 0 μs; a, c, e, g, i, k, m) and final (*t* = 10 μs; b, d, f, h, j, l, n, o, p) snapshots of the 16 copies of M2TM or M2TM-AH (backbone beads in pink) in lipid bilayers (phospholipid PO4 beads in green) with a Martini force field.^64,71−73^ The snapshots show (a, b) M2TM in a DMPC; (c, d) M2TM in POPC; (e, f) M2TM in POPC/cholesterol; (g, h) M2TM-AH in DMPC; (i, j) M2TM-AH in POPC; (k, l) M2TM-AH in POPC/POPS; (m, n) M2TM-AH in POPC/POPS/Chol; (o) snapshot at *t* = 10 μs of M2TM-AH in POPC/cholesterol showing the membrane curvature surrounding the pentameric species; (p) top view of the M2TM-AH pentamer captured POPC/cholesterol, shown in panel (o). Each panel demonstrates a clipped view of the full 16-protein copy system. These figures do not represent the full systems, presenting only a selected “slice” of the system shown.

The shape formation of M2 channel clusters is illustrated in Figure 2, which shows “slices” of the 16 copy-M2 channel system from initial (t = 0 μs; Figure 2a,c,e,g,i,k,m) and final (t = 10 μs; Figure 2b,d,f or o,h,j,l,n) simulation snapshots from CG MD simulations of seven systems in membranes consisting of either multimers of M2TM channels in DMPC (Figure 2a,b), POPC (Figure 2c,d) or POPC/cholesterol bilayers (Figure 2e,f), or multimers of M2TM-AH channels in DMPC (Figure 2g,h), POPC (Figure 2i,j) or POPC/cholesterol bilayers (Figure 2k,l). The largest M2TM clusters consisting of four M2TM channels were oriented such that their pore axis was approximately parallel to the membrane normal (Figure 2b, Figure S2). The dimers of M2TM-AH channels were more conical-shaped (see, e.g., Figure 2h); thus, the individual channels’ axes were tilted from the membrane normal (Figure 2h, Figure S2). Interestingly, regarding the pentamer assembly of M2TM-AH in POPC/cholesterol, every M2 protomer interacted with a maximum of two different M2 channels (linear clustering^24^). This was what we observed in all the clusters of M2TM-AH channels and is likely due to M2TM-AH’s conical shape. Upon the formation of channel dimers, the M2TM-AH channels were osculated to one another, and their pore axes became tilted to one another. As a result, there was only one binding site for an additional M2 channel. Once a conical-shaped dimer of M2TM-AH channels was formed, the protein channels generally remained clustered almost throughout the simulation.

### Protein–Protein Interactions

M2TM-AH channels in the clusters appeared to interact with each other through the residues of the AHs and the N-terminal regions, suggesting that these specific PPIs play a significant role in cluster stabilization. These findings agree with the previously reported observations in CG MD simulations of M2TM^28^ and M2TM-AH channels^24,28^ and with ssNMR results using ^19^F labels.^31^

**Figure 3:**
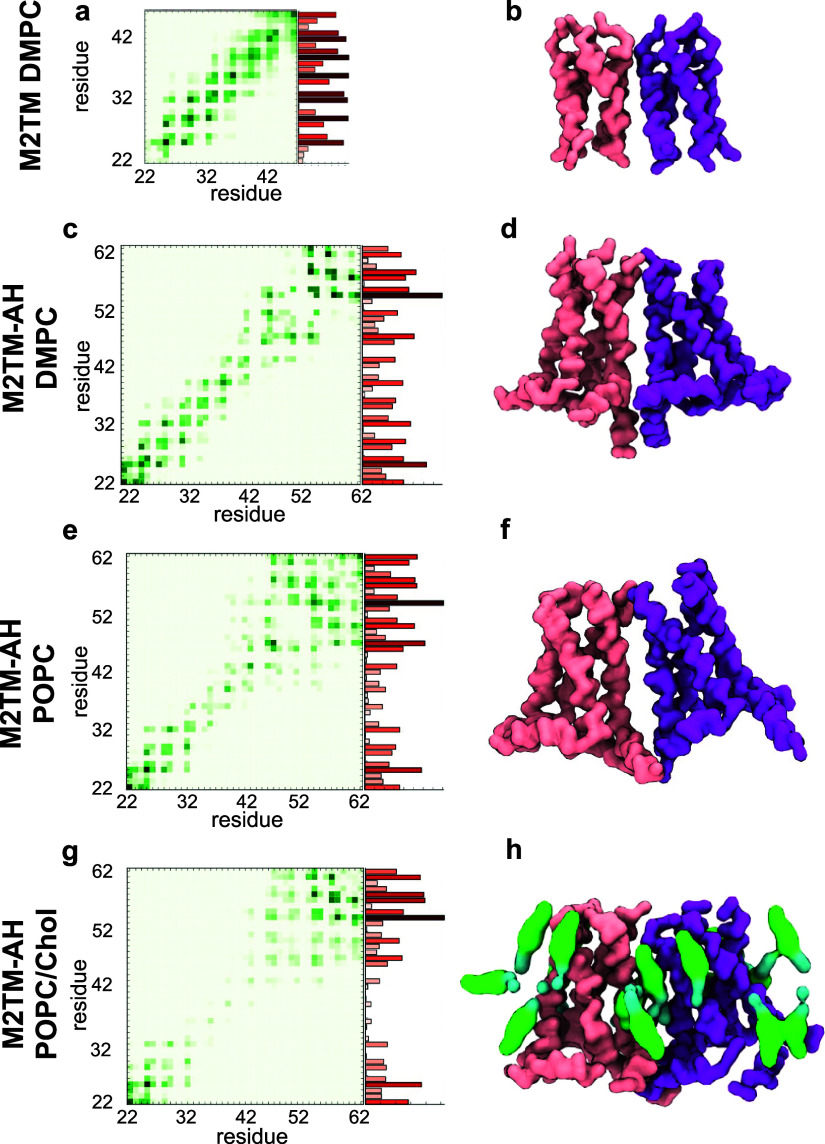
M2TM and M2TM-AH protein–protein interaction (PPI) analysis. The PPIs are presented as interprotomer heatmaps and histograms integrated across the residues of the M2 constructs. Data is extracted from 2 × 10 μs CG MD simulations with Martini force field^64,72,73^ of 16 M2 construct copies. (a, c, e, g) Heatmaps correspond to the pairwise interaction frequencies between residues in the adjacent M2 channels shown in (b, d, f, h) using a green scale from light green (lower frequency of interactions) to dark green (higher frequency). Histograms show the total residue interaction frequency (averaged across all four peptide chains in an M2 channel) using a red scale from light red (lower) to dark red (higher). The representative M2 dimer configurations shown were taken from the last frame of one of the repeat simulations.

We quantified the specific PPIs responsible for clustering M2 channels by plotting the frequency of the contacts between amino acid residues belonging to different channels. The analysis included all M2 clusters from replicate simulations. For the M2TM channel clusters, the contacts between two adjacent channels included predominantly hydrophobic residues throughout the length of the TM domain (Figure 3a,b). In contrast, for the M2TM-AH channel clusters (Figure 3c–h), the PPIs between adjacent channels corresponded to contacts of polar residues at the N-termini of TM helices and contacts of residues at the C-termini between AHs.^30−32^ Specifically, the residues with the highest interaction frequency between M2TM-AH–M2TM-AH channels in a multimer were S22 and P25 at the N-termini and the F54, H57, G58, and R61 of the AHs (Figure 3c,e,g) compared to the residues located at the transmembrane core (residues 32 to 48). When we compared these PPIs between M2TM-AH multimers in POPC (Figure 3e,f) and M2TM-AH multimers in POPC/cholesterol (Figure 3g,h), a considerable reduction in the interaction frequencies between TM residues in different protomers was observed in the latter system (Figure 3e), suggesting cholesterol is involved in the clustering.

Thus, our CG MD simulations suggested that cholesterol molecules are distributed between the TM cores of adjacent channels. We hypothesized that this can cause either weakening of multimer formation, or that cholesterol can act as a bridge between M2TM-AH protomers, potentially as an auxiliary mediator. The latter can explain the larger cluster ranks observed in cholesterol-containing membranes (Figure 1).

### Protein–Lipid Interactions

**Figure 4:**
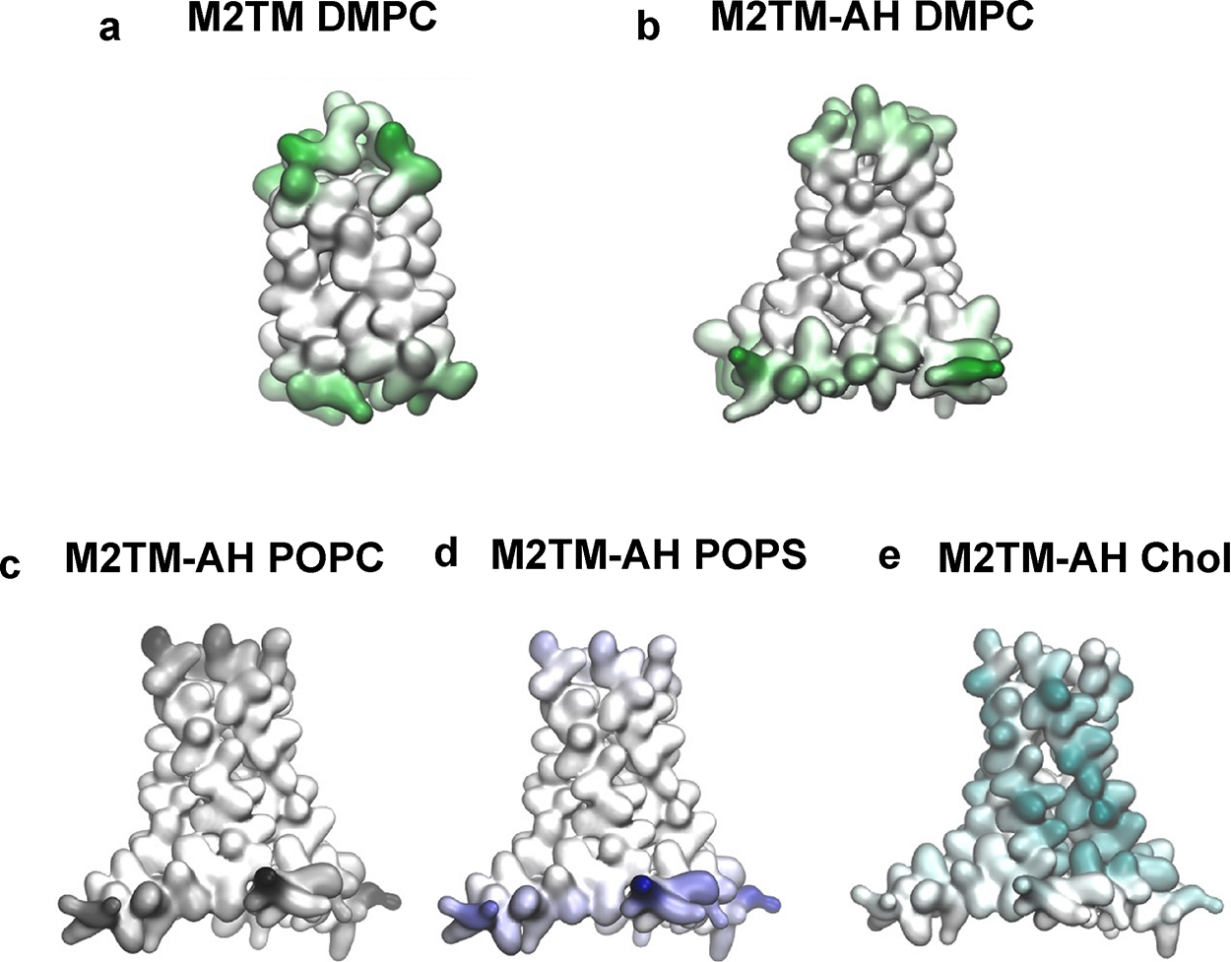
M2TM and M2TM-AH protein–lipid interaction analysis. Lipid interaction heatmaps projected onto CG surface representations of M2TM and M2TM-AH averaged over 2 × 10 μs and over the 16 independent protein copies per simulated system. Each panel shows data for the indicated lipid type, accounting for interactions with the phospholipid headgroup (PO4 beads) or cholesterol. (a) M2TM with DMPC lipid headgroups; (b) M2TM-AH with DMPC lipid headgroups; (c) M2TM-AH with POPC lipid headgroups; (d) M2TM-AH with POPS lipid headgroups; (e) M2TM-AH with cholesterol. For the M2TM–DMPC interactions, the M2TM–DMPC system was analyzed. The heatmaps for M2 are colored according to the interacting lipid; green for DMPC polar heads in panels (a) and (b); gray for POPC polar heads in panel (c); blue for POPS polar heads in panel (d); teal for cholesterol in panel (e).

To further test cholesterol's role in mediating clustering, we explored M2’s interaction profile with the bulk lipid environment (Figure 4; Figure S3). Figure 4 shows heatmaps of contacts between M2 channel residues and the phospholipid headgroups or cholesterol plotted from CG MD simulations considering all of the multimeric states of M2.

In the case of M2TM–DMPC or M2TM-AH–DMPC interactions, we analyzed the M2TM–DMPC or M2TM-AH–DMPC systems, respectively. For the M2TM-AH–POPC, M2TM-AH–POPS, and M2TM-AH–cholesterol interactions, we analyzed the M2TM-AH–POPC/POPS/cholesterol system. The analysis showed that DMPC and POPC polar heads interacted with the same M2 residues. Figure S3 shows frequency interactions of the M2 residues that interact with the different lipid headgroups. M2TM and M2TM-AH span the DMPC or POPC bilayer and interact primarily through the same interfacial residues S22, S23, and D24 with the polar head region of the top leaflet, and through D44, R45, L46 (M2TM) and S50, R53, H57, R61 (M2TM-AH) with the bottom leaflet (Figure 4a–c; Figure S3a–c).^65^ In the case of POPS, the negatively charged headgroup did not interact extensively with the N-terminal end of M2, which contains the negatively charged D24 residue, showing marked preference to interact via the bottom leaflet (Figure 4d; Figure S 3d). For the M2TM-AH in POPC/cholesterol and POPC/POPS/cholesterol systems, the interactions between M2TM-AH and bulk cholesterol included the whole stretch of the lipid-facing protein residues (Figure 4e; Figure S3e), with the highest frequency recorded for P25. In the following section, we further analyze the interactions between M2TM-AH and cholesterol. However, the difference in contact frequency between protein and DMPC, POPC, and POPS phospholipid head groups (Figure 4a–d) and that between protein and cholesterol (Figure 4e) was not surprising because cholesterol extends deep into the membrane interior and can contact buried residues, several at a time. In contrast, the phospholipid head groups spend most of their time in the interfacial region.

### Protein–Cholesterol Interactions

**Figure 5:**
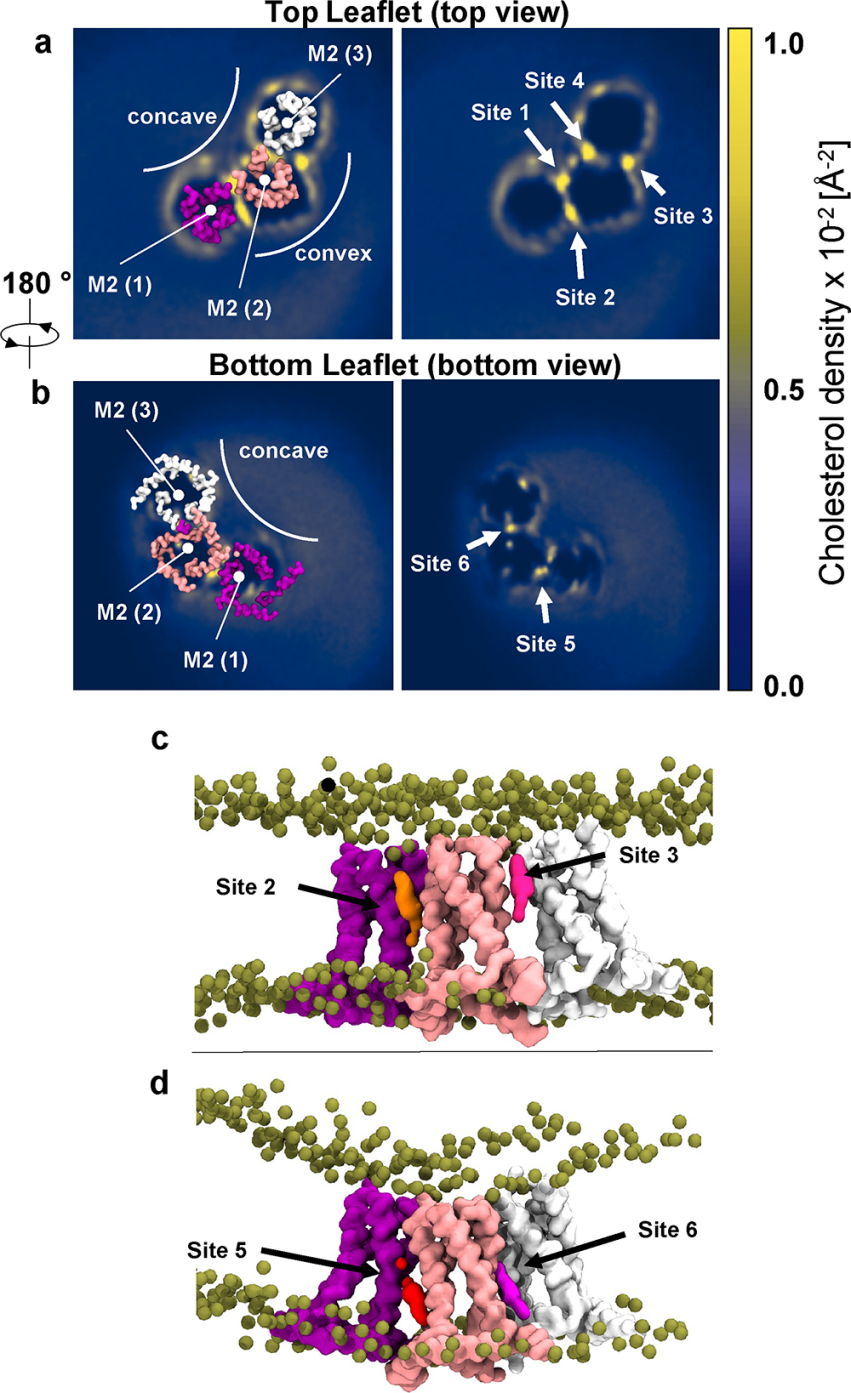
Cholesterol enrichment in the M2TM-AH annulus. 2D time-averaged occupancy of cholesterol shown as membrane-plane projections from 2 × 50 μs CG MD simulations of an M2TM-AH trimer in POPC/cholesterol (1 Å resolution grid was used). The three M2TM-AH protomers were numbered as M2(1), M2(2) and M2(3). (a) Top view showing the high density–low residency sites 1–4. (b) Bottom view showing high density–high residency sites 5, 6. Normalized cholesterol density (in Å^–2^) ranges from blue (low values) to yellow (high values). (c) Side view of a simulation snapshot showing top leaflet cholesterols binding to sites 2 and 3. (d) Side view of a simulation snapshot showing bottom leaflet cholesterols binding to sites 5 and 6. Proteins are represented as CG surfaces colored purple, salmon, and white (M2(1), M2(2), and M2(3), respectively).

Our CG MD simulations suggested that in POPC/cholesterol and POPC/POPS/cholesterol systems cholesterol is distributed between the TM cores of adjacent M2TM-AH channels. The M2TM-AH channel clusters formed include cholesterol molecules between the protomers. To further investigate the equilibrium stability of the clusters formed and their interfacial cholesterols, a membrane patch with a trimer of M2TM-AH channels was extracted from the POPC/cholesterol system and subjected to a further 2 × 50 μs MD. During the simulations, this cluster remained a stable trimer, and cholesterols continued to bridge adjacent M2 protomers (Figure 5). Calculating the time-averaged cholesterol density revealed six topologies with pronounced cholesterol density or, equivalently, three binding sites per degenerate pair of M2 channels (the trimer has C2 symmetry) (Figure 5a,b). The cholesterol binding sites are shown in Figure 5c,d. Specifically, we report two sites 1, 2 (and their symmetrical equivalents, 4 and 3) bridging two protomers in the top membrane leaflet. Another cholesterol binding site, 5 (and its symmetrical equivalent, 6) (Figure 5b,d), is in the bottom membrane leaflet.

**Figure 6:**
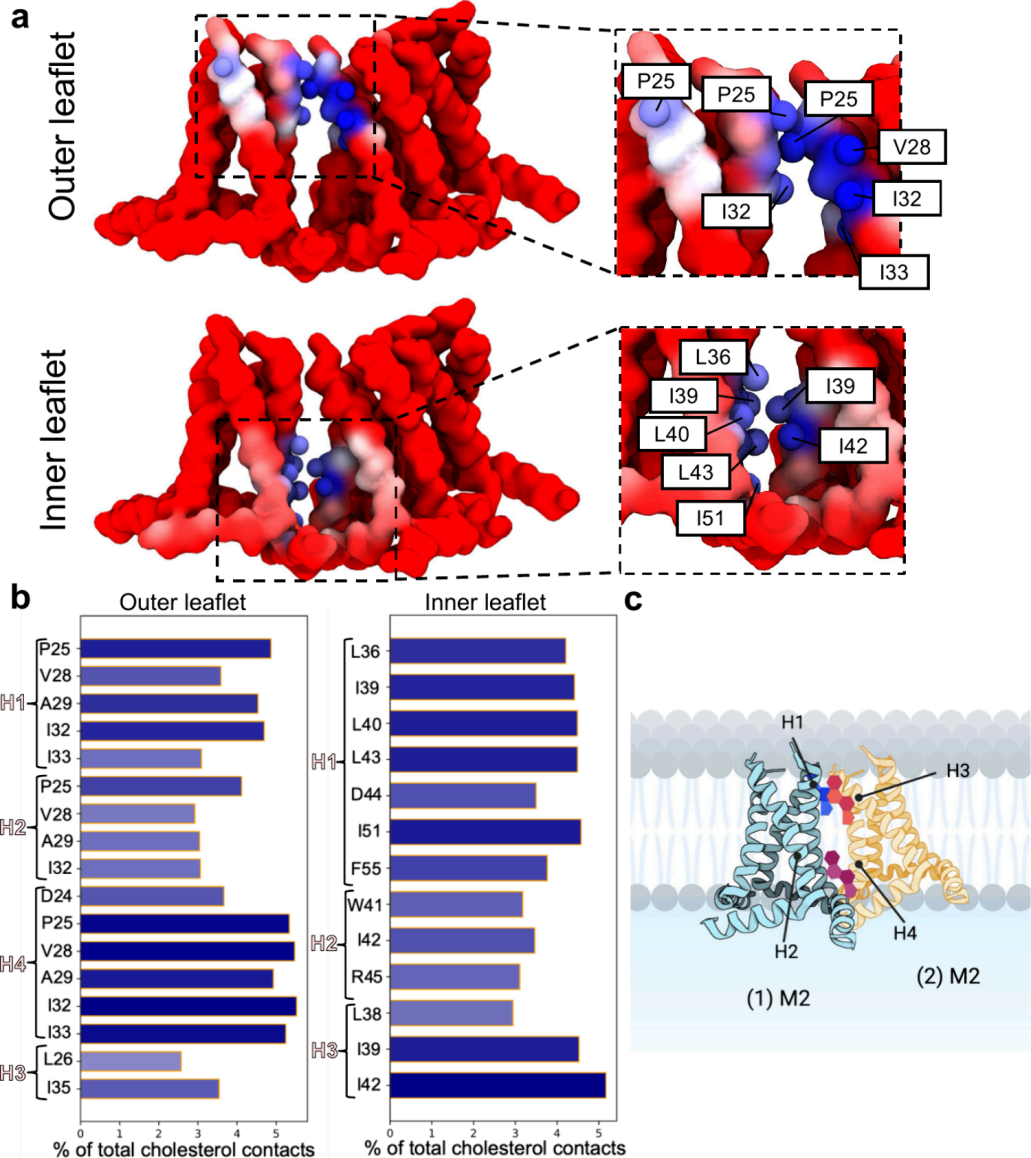
M2TM-AH cholesterol interaction and hotspot analysis. (a) Side view of a snapshot from the 50 μs CG MD simulation of the M2TM-AH trimer, with protomers numbered as in Figure 5. Time-averaged interactions between M2 (1), M2 (2), and top and bottom leaflet cholesterols are analyzed. M2(1) and M2(2) residues are colored according to their interaction frequency with cholesterols, using a red (low) to blue (high) coloring scale. The residues with their side chain beads in dark blue correspond to those with the most frequent interactions (>4% of total cholesterol contacts). (b) Plots of the most frequent residue interactions from the two M2TM-AH protomers that have contacts with the top and bottom leaflet cholesterols. Contacts are calculated using a 6 Å distance cutoff to define a “contact” based on the radial distribution function for CG Martini^64,72,73^ lipid–protein interactions. (c) Schematic representation of the analysis showing two M2 protomers in (a) with their interfacial top and bottom leaflet sites bound by cholesterol. The numbered helices (H) include H1 and H2 from M2 (1) and H3 and H4 from M2 (2).

We hypothesized that the aforementioned cholesterol densities might form discrete cholesterol binding sites that stabilize the trimer and, by extension, the M2 clusters, which appeared to be enriched in the presence of cholesterol. For clarity, in Figure 6, we assign the three M2TM-AH protomers of the trimer as M2(1), M2(2), and M2(3) and present a cartoon schematic of the topology between two M2 protomers M2(1), M2(2) is depicted. Protein–lipid interaction analysis showed that specific residues in the TM α-helices 1–4 (H1–H4) that line the interprotomer space interact with membrane cholesterols (Figure 6a,b). These residues could be grouped in those interacting with top and bottom leaflet cholesterols. The four top leaflet cholesterol binding sites of the full trimer (1, 2, or 4, 3) interacted with the N-terminal end of TM in the two channels. Specifically, the per residue interaction analysis showed that in the top leaflet, H1’s high-frequency interactions involved residues P25, A29, and I32, whereas H4 mostly interacted with cholesterol through residues P25, V28, A29, I32, and I33 (Figure 6a,b). Table S2 shows the extracted kinetic parameters for the three unique cholesterol binding sites for each pair of M2TM-AH protomers calculated using PyLipID.^74^ Despite the high occupancy of the four top leaflet cholesterol binding sites (sites 1–4; > 65%), cholesterols showed lower residence time compared to the bottom leaflet sites (sites 5 and 6), suggesting that the bottom leaflet sites have a slower *off*-rate compared to those of the top leaflet. Surprisingly, this analysis showed different cholesterol binding kinetics between the degenerate pair top leaflet sites 1/4 and 2/3. Specifically, sites 1 and 4 have residency of 4.7 μs, occupancy of 67%, and site surface of 7.7 nm^2^, whereas sites 2 and 3 have residency of 9.8 μs, occupancy of 92%, and site surface of 17.1 nm^2^ (Table S2). A reduced site surface area is in line with reduced cholesterol accessibility in sites 1 and 4 compared to those in sites 2 and 3 (Figure 5a), highlighting that top-leaflet cholesterol is preferentially stabilized in the convex side of the trimer.

The bottom leaflet sites (sites 5 and 6; Figure 6a) captured cholesterols into specific interprotein binding clefts surrounded primarily by hydrophobic residues with high time-resolved occupancy (88.5%) and high residence time (15.4 μs) (Table S2). This increased cholesterol residency in the bottom leaflet suggested tighter, more specific interactions. The bottom leaflet residues with high cholesterol interaction frequency are L36, I39, L40, L43, and I51 from H1 of the first M2 channel and I39 and I42 from H3 of the second M2 channel (Figure 6a,b). Cholesterol-interacting residues were reported in previous ssNMR experiments,^55−57^ which showed cholesterol binding close to the AH and in contact with residues I39, I42, and F47. We previously captured these residues computationally on a single M2TM-AH, showing cholesterol binding (a) in the N-terminal interhelical cleft and (b) between the TM and AH domains (top and bottom leaflets, respectively) with unbiased atomistic simulations and binding kinetics analysis.^65^ While the lower-residency sites 1 and 4 are on the concave trimer surface, sites 2, 3, and 5, 6—associated with higher cholesterol residency times—are on the convex surface (Figure 5a,b).

**Figure 7:**
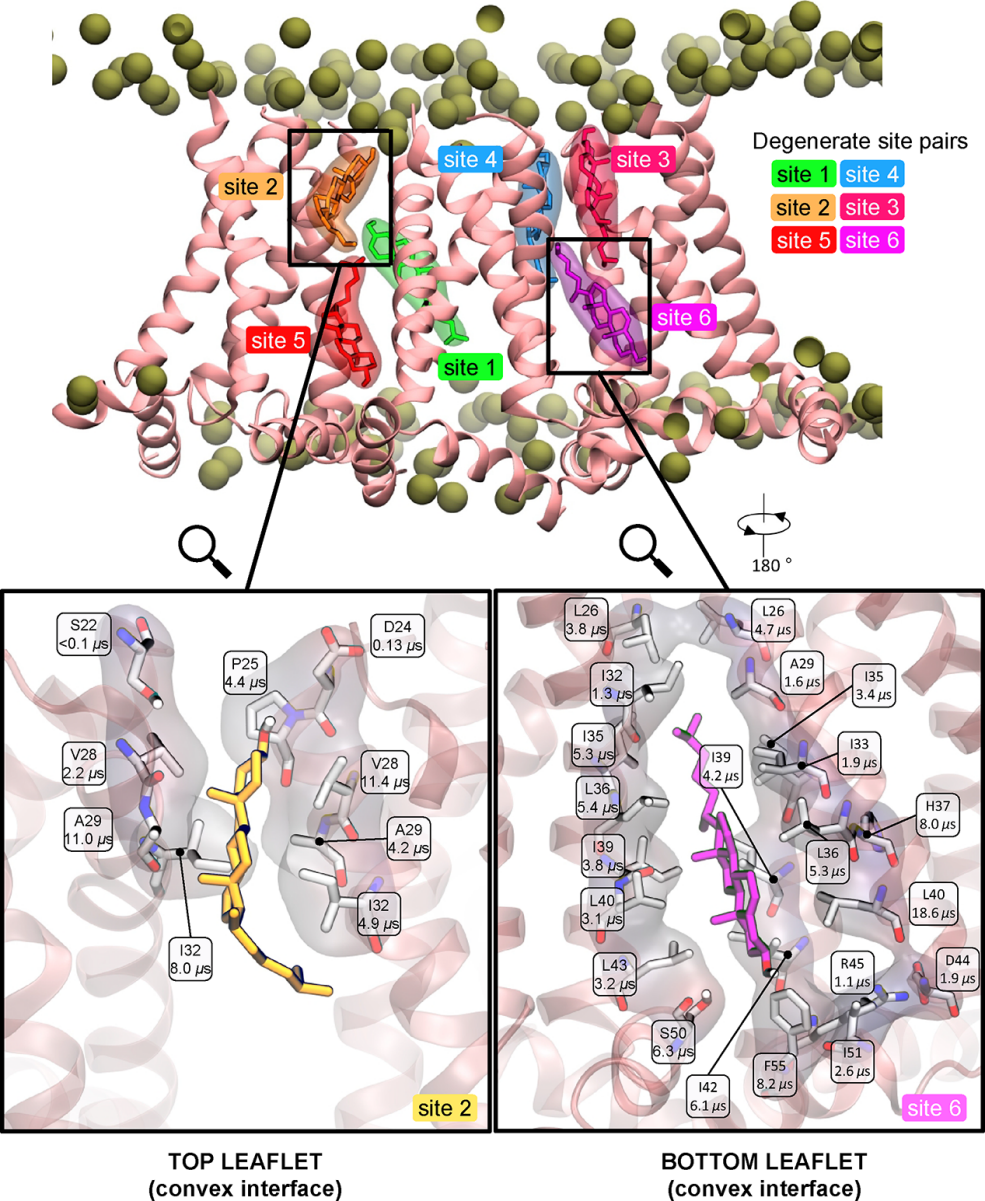
Representative binding configuration of interfacial cholesterols on both leaflets, top (binding sites 1, 2, 3, 4) and bottom (binding sites 5, 6) in 4:1 POPC:cholesterol from 2 × 50 μs CG MD simulations. The binding configurations were analyzed with the PyLipID python package (see Methods). The binding configuration shown has all high-residency sites occupied (defined as those with overall site residence time >1.0 μs). Zoomed-in views show the cholesterol-interacting protein residues in white stick representation and a transparent off-white van der Waals surface from binding sites 2 (left) and 6 (right). The per-residue residency time (calculated as 1/*k*_*off*_, see Methods) is shown. M2 cartoon helices are shown in salmon.

To quantitatively weigh the respective contributions of residues coordinating cholesterols at the convex interface, we deconstructed the reported binding sites to their residue components and calculated the per residue *off*-rates over 2 × 50 μs (Figure 7). In the top leaflet, cholesterols form hydrogen bonds with S22 (<0.1 μs) and D24 (0.13 μs) and are accommodated in the lipophilic cleft by van der Waals interactions with P25 (4.4 μs), A29 (11.0, 4.2 μs), I32 (8.0, 4.9 μs), and V28 (11.4, 2.2 μs). The increased residence time observed for the hydrophobic residues suggests that the pocket behaves as a “greasy” patch, as suggested by others for cholesterol pockets,^75,76^ without a specific, high-affinity residue. Similarly, in the bottom leaflet, we find cholesterol is engulfed by an extensive lipophilic pocket with the longest, most-stabilizing interactions including TM residues L26 (3.8, 4.7 μs), I35 (5.3, 3.4 μs), L36 (5.3, 5.4 μs), H37 (8.0 μs), I39 (4.2, 3.8 μs), L40 (18.6, 3.1 μs), and I42 (6.1 μs). In contrast to the top leaflet site, a more extensive network of hydrophilic interactions coordinates the polar hydroxyl group in the bottom leaflet. Specifically, D44 (1.9 μs) and R45 (1.1 μs), but primarily AH residues S50 (6.3 μs), I51 (2.6 μs), and F55 (8.2 μs) are involved. The residues marked with high binding residency times (Figure 7) agree with those with high interaction frequencies averaged over all equivalent M2 residues (Figure 6b), revealing that the interactions are not due to transient, short-lived cholesterol contacts with M2 but reflect stable binding. Collectively, the analysis suggests that an extended membrane-facing residue network lines the cholesterol sites. Most importantly, while these residues can make transient interactions with cholesterol in monomeric channels,^65^ upon clustering they create “greasy” pockets for steroid group stabilization with more polar residues serving to auxiliary stabilize the hydroxyl group.

### Energetics of Cholesterol-Mediated M2TM-AH Channel Dimerization

Driven by the above results, which demonstrated increased multimeric clustering of M2TM-AH in the presence of cholesterol and that cholesterol has well-defined interfacial binding sites between clustered M2TM-AH protomers, we proceeded to quantify the relative role of cholesterol in the M2TM-AH dimerization free energy profiles. Dimerization free energies for membrane proteins are computationally demanding and thus scarce in the literature.^77−81^ We used an M2TM-AH dimer with three cholesterols between the protomer pairs (in binding sites 1, 4, and 5) from the simulation in POPC/cholesterol bilayers (see Methods). We also considered an equivalent control M2TM-AH dimer lacking cholesterol isolated from the simulations in POPC bilayers. Both dimers (±cholesterols) were embedded and equilibrated in their bilayers, and their stability was assessed with the CHARMM36m force field^82^ to ensure both configurations were stable with an atomistic force field. We verified both dimer configurations to be stable over 3 × 500 ns repeats by tracking the interprotomer distance and rotation of one protomer relative to the other over time (Figure S9). Thus, we proceeded with CG MD PMF(US) calculations.

**Figure 8:**
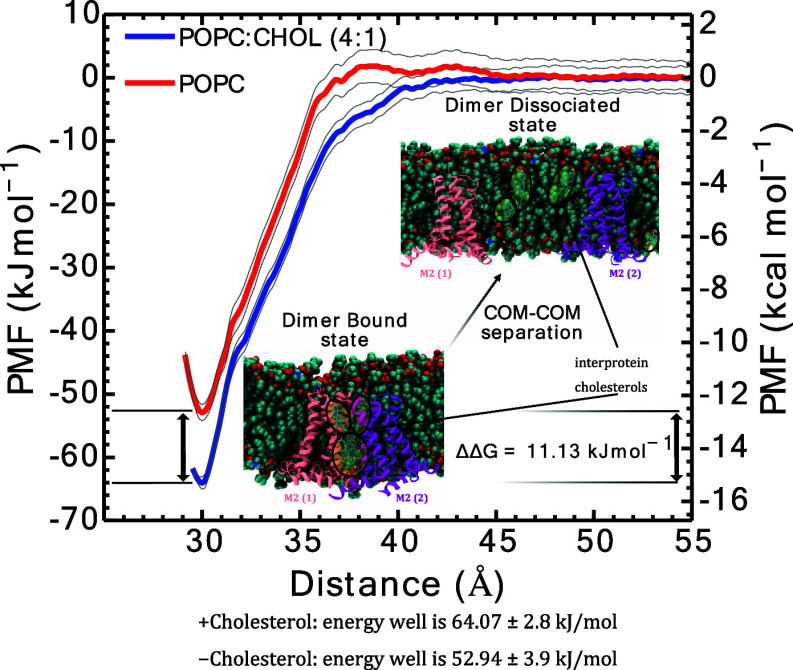
M2TM-AH dimerization PMF profiles (±cholesterols) from CG MD simulations with the Martini force field,^64,72,73^ analyzed with WHAM.^84,85^ The PMFs were calculated by dissociating the M2TM-AH protomers from dimers isolated from the larger 16-copy systems in POPC or POPC/cholesterol (4:1). The dimer dissociation into M2TM-AH protomers was driven by linearly increasing the distance between the protomers’ COM on the membrane *xy* plane at a constant rate 0.0001 nm/ns and with a force constant 1000 kJ/mol/nm^2^. The COM distance collective variable ranged from 30.0 to 56.0 Å, and system replicas were selected every 0.5 Å, for a total of 54 windows per system. For the US windows, the force constant was 10 kJ/mol/nm^2^ for 3–4.5 Å and 1000 kJ/mol/nm^2^ for 4.5–5.6 Å. Each US window was equilibrated for 200 ns in NPT followed by 4.8 μs production time, for a total of 518.4 μs window sampling time (see Figures S4 and S5). The calculated free energy well with cholesterol was −64.07 ± 2.8 kJ/mol, and without cholesterol, it was −52.94 ± 3.9 kJ/mol.

We report both M2TM-AH dimerization free energies (±cholesterols) as PMF(US) profiles calculated using the previously applied weak orientational restraint method to preserve the orientation of one protomer relative to the other^83^ (see Methods Section) (Figure 8). Satisfactory US window overlap along the interprotomer distance CV was achieved by inspecting histogram overlap (Figures S4 and S5) and increasing the umbrella force constant/reducing window spacing where necessary (see Methods). The dissociation of the dimer of M2TM-AH channels into protomers was driven by linearly increasing the *xy* plane distance between the protomers’ center-of-mass (COM) from 30.0 to 56.0 Å. Each US window was equilibrated for 200 ns in the NPT ensemble followed by a 4.8 μs production time, for a total of 518.4 μs window sampling time.

The PMF landscapes show that within the distance between the COM ranges of 29–31 Å, the same local minimum is observed for both systems (Figure 8). The dimerization free energy of M2TM-AH protomers without cholesterol was −52.94 ± 3.9 kJ/mol (−12.66 ± 0.9 kcal/mol). The dimerization free energy of M2TM-AH protomers with the three interprotein cholesterol sites occupied was −64.07 ± 2.8 kJ/mol (−15.31 ± 0.7 kcal/mol). Thus, the PMF(US) calculations revealed that cholesterol strengthens the free energy of association for M2TM-AH assemblies by ΔΔ*G*_POPC→POPC:cholesterol_ = −11.13 kJ/mol (−2.65 kcal/mol). Since ΔΔ*G* is logarithmically related to the ratio , this translates to the M2TM-AH–M2TM-AH dimer state being almost 2 orders of magnitude more populated when the interprotein sites are occupied by cholesterol. This result agrees with our observation from unbiased MD of increased M2 cluster sizes upon addition of 20 mol % cholesterol in the membrane (Figure 1b,d). Cholesterol binds between two adjacent M2TM-AH protomers in a dimer at sites with high occupancy and slow *off*-rates. Collectively, this suggests that cholesterol acts as a molecular glue by strengthening M2TM-AH association via specific protein-cholesterol-protein bridges, thus enhancing multimer formation.

We also note that the PMFs agree with the out-of-equilibrium time-dependent oligomer tracking presented in Figure 1 for POPC vs POPC/cholesterol. Specifically, the increased dimerization energy of M2TM-AH when the interfacial pockets are occupied with cholesterol (Figure 8) qualitatively agrees with the higher oligomerization speed observed when cholesterol is present in the membrane (Figure 1b,c).

### M2AH-Cholesterol Complexes Influence Membrane Curvature

The AHs of the M2 channel mediate the membrane undulations^24−31,49^ that are necessary for the membrane-curvature mechanism of virus budding.^32−34,36,37,26−33,51^ While multiple single point mutations in the AHs did not affect viral replication, suggesting that specific residues are not critical,^86^ the penta-Ala M2 construct, where five bulky hydrophobic residues on the AH are replaced with alanines, is known to promote significantly reduced negative Gaussian curvature.^26^ Furthermore, previous studies suggest that cholesterol enhances the ability of M2TM-AH to generate membrane curvature.^24−31,49^ We show above that the clustering of M2TM-AH enhances the deformation of planar membranes during unbiased MD simulations (Figure 2). By extension, we focused on quantifying the curvature produced by M2TM-AH in our model membranes and on whether this is affected by the absence of the AHs or cholesterol. An initial analysis showed that time-averaged local curvature canceled out over time (partly due to the diffusive behavior of proteins in bilayers), meaning that we could not use the average membrane curvature across the simulation time as a metric. However, visual inspection of the trajectories suggested differential membrane curving between M2TM-AH, M2TM, and protein-free membranes (Figure S10a–j). Membrane deformation along the *z*-axis (membrane normal) showed that M2TM-AH clusters are populated in membrane valleys (as seen from a top view, Figure S10b,f,g,h), whereas M2TM can cluster outside valleys as well (Figure S10a,c,d). We note that while a protein-free POPC bilayer remained relatively planar (Figure S10i), the addition of cholesterol caused spontaneous deformations (Figure S10j), as also seen in a recent study where cholesterol was found to be enriched in areas of increased curvature.^87^

**Figure 9:**
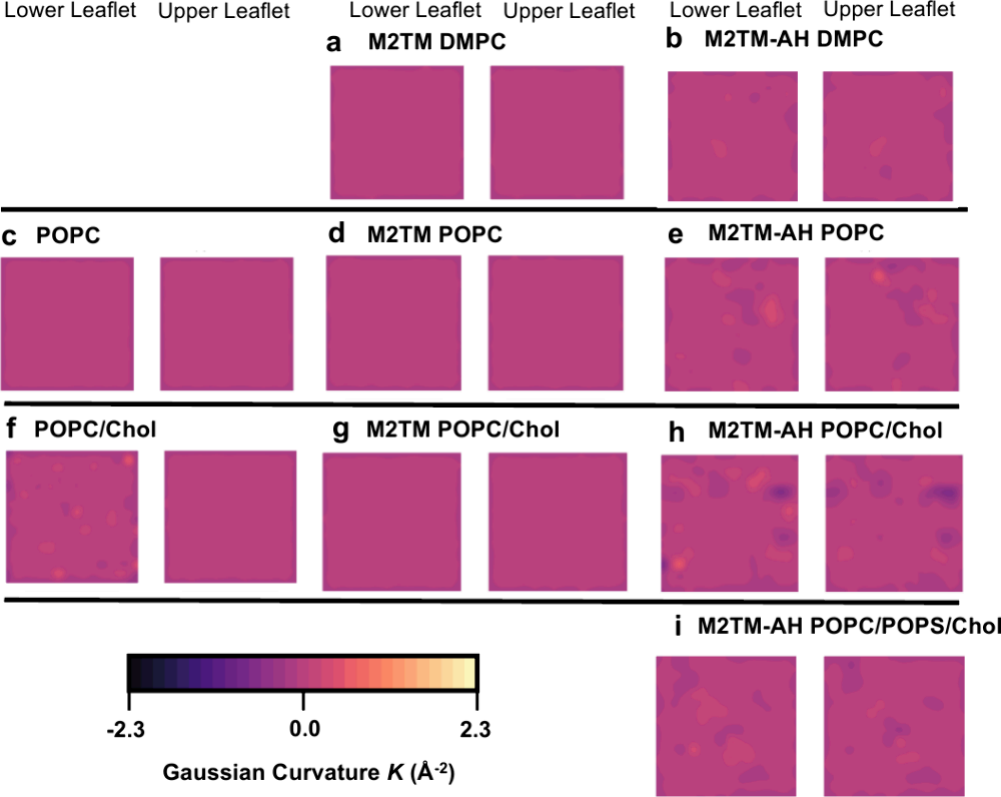
Gaussian curvature (*K*) induced by M2 constructs (±AHs) and cholesterol from 10 μs CG MD of 16 copies of the M2 constructs and protein-free control bilayers with the Martini force field.^64,71−73^ The *K* heatmaps are averaged over the final 2 μs from a single replicate. Simulated systems are shown as follows: (a) M2TM in DMPC; (b) M2TM-AH in DMPC; (c) POPC; (d) M2TM in POPC; (e) M2TM-AH in POPC; (f) POPC/cholesterol; (g) M2TM in POPC/cholesterol; (h) M2TM-AH in POPC/cholesterol; (i) M2TM-AH in POPC/POPS/cholesterol. *K* is shown in a color gradient ranging from black to bright yellow (−2.3 to 2.3 Å^–2^). Results for the second replicate are shown in Figure S11. Averaging over shorter simulation intervals highlighted the quicker temporally resolved *K* fluctuations. Examples over the last 100 ns are shown in Figures S12 and S13.

We hypothesized that clusters of M2TM-AH channels could exert a linactant deformation force on annular lipids and, via surface tension modulation, regulate curvature. To quantify M2’s effect on membrane planarity, we calculated the Gaussian curvature (*K*) on a surface fitted to the membrane, z_*ij*_ (*i* and *j* define grid cells (*i,j*)), defined as *K* = ∂*_xx_*∂*_yy_* – ∂_*xy*_^2^/(1 + ∂_*x*_^2^ + ∂_*y*_^2^)^2^, and projected on the membrane *xy* plane (Figure 9a–i; Figure S6). The data were averaged over the final 2 μs of the simulations as longer averaging times resulted in the “flattening” of the landscapes due to undulations traversing the *xy* plane. *K* > 0 means a spherical membrane protrusion (on either side of the membrane, i.e., hills or valleys), whereas *K* < 0 refers to a saddle-shaped hyperboloid. We also tracked the time evolution of the absolute net Gaussian curvature (|*K*|) over the whole simulation (10 μs) as a measure of total global membrane curving (Figure S7). Equivalent *K* heatmaps resulting from the second set of simulation repeats are shown in Figure S11. Both repeats were averaged and shown in scatter dot plots (Figure S6), and statistics are tabulated in Table S4. Additionally, *K* heatmaps averaged over only the final 100 ns of the repeats accentuate the temporally resolved membrane undulations (Figures S12 and S13).

The Gaussian curvature analysis showed that when AHs are present, local membrane curvatures are enhanced compared to the curving observed in M2TM systems lacking AHs (Figure 9). M2TM-AH increased the Gaussian curvature range of a POPC bilayer (protein-free; −0.221 to 0.035 Å^–2^, +M2TM; −0.253 to 0.034, +M2TM-AH; −0.302 to 0.577 Å^–2^) (Table S4). Interestingly, the AHs did not however exert such a pronounced effect on a stiffer DMPC membrane (+M2TM; −0.272 to 0.296, +M2TM-AH; −0.241 to 0.211 Å^–2^), suggesting that M2TM-AH’s curvature induction is limited by the bending modulus (25.3 and 34.7 *k*_B_*T* for POPC and DMPC, respectively^88^). We also find that cholesterol increased the global curvature in both leaflets when added in protein-free bilayers (protein-free POPC; −0.221 to 0.035 Å^–2^, protein-free POPC/cholesterol; −0.570 to 0.589 Å^–2^), in agreement with ssNMR results^89^ and CG MD simulations reported by others.^87^ Adding M2TM-AH in POPC/cholesterol induced further negative and positive *K* in both leaflets (Figure 9h) and produced the largest *K* range among all the systems compared (+M2TM-AH; −1.357 to 0.952 Å^–2^). Notably, the AHs coupled with cholesterol produced the most negative *K*, the curvature type topologically necessary for a number of membrane destabilization events, including membrane budding and scission. Removing the AHs only modestly increased system-wide curving (M2TM/POPC/cholesterol; −0.245 to 0.046 Å^–2^, M2TM/POPC; −0.253 to 0.034 Å^–2^), suggesting that cholesterol’s curvature inducing effects are mitigated in the presence of just the TM domain. Our result highlights that spontaneous saddle-shaped patches can be formed without external bending forces when both AHs and cholesterol components are present.

The quantitative comparisons presented here suggest an additive, synergistic mechanism between the AHs and cholesterol, consistent with their roles in increased clustering. (Figure 1c). Clustering in POPC/cholesterol membranes is localized in curved membrane patches, where *K* is nonzero (Figure 2o; Figure S10f). Indeed, in POPC/cholesterol membranes M2TM-AH showed the largest channel cluster sizes (reaching up to pentamers) and the most stable in time clusters (Figure 1c), with trimers forming within the first μs of the simulation repeats. From a membrane biology standpoint, this suggests that the AHs are essential to navigate M2 channel clusters to curved membrane patches where cholesterol is enriched^87^ or, vice versa, that the AHs are essential for the clusters to induce local curvature.

The addition of the anionic POPS in the M2TM-AH/POPC/cholesterol mixture suppressed negative Gaussian curvature (Figure 9j, +POPS; −0.322 to 1.046 Å^–2^, -POPS; −1.357 to 0.952 Å^–2^). Despite the reduction in the *K* range, M2TM-AH forms higher-order oligomers in the presence of POPS (>2 channels; Figure 1e). To better understand this, we tested whether the interfacial cholesterol binding sites we discussed above (see protein–cholesterol interactions) can still bind in the presence of POPS. Indeed, when we embedded and simulated the isolated trimer in a membrane that includes both cholesterol and POPS, the interfacial cholesterol sites were still identified (Table S3). The analysis showed residency times >1.5 μs and occupancies >50% with the addition of POPS, showing that interfacial cholesterol can bind the same sites. However, we noted decreased binding residency times for the interfacial sites (see Table S2 versus Table S3). We wondered whether this might be due to a direct POPS competition effect for the interfacial sites. However, no interprotein POPS binding sites were found. A single noninterfacial, peripheral POPS binding site was found with lower residency time (0.84 μs) and low occupancy (32.6%) over three simulation repeats, suggesting that the reduction in cholesterol binding observed is noncompetitive. Other POPS hotspots showed <0.1 μs binding times and <25% occupancies, indicating that compared to cholesterol, POPS is dynamically mobile with distinct POPS molecules exchanging rapidly. Thus, POPS reduces *K* and exerts a noncompetitive reduction in interfacial cholesterol binding (in the concentration used, 20% mol, i.e., 1:1 mol with cholesterol). Notably, POPC/POPS without cholesterol also resulted in transient M2TM-AH cluster formation/deformation over time (Figure 1d), which we did not observe in the other lipid systems.^87,89^

## Discussion

For many enveloped viruses, including HIV and influenza, assembly and budding occur from membrane microdomains (e.g., lipid rafts)^37,90^ to form virions. Understanding the mechanisms underlying this process could promote biomedical efforts to block viral propagation.

M2 preferentially localizes at the edge of membrane lipid rafts,^32−34,36,37,42,52^ which develops into the bud neck position (also referred to as the budozone).^32−37^ Such localization of a fission-inducing protein at the neck of the budding virus near lipid rafts^52,53^ causes membrane undulations in model planar membranes.^24−31,49^ Others have previously suggested the formation of clusters of influenza A M2 channels in the saddle-shaped budding neck, prior to cell membrane scission, implicating possible involvement of M2 clustering in the viral budding mechanism.^24−34,36,37^ While the AHs of M2 were shown to contribute to cluster formation and membrane undulations in planar plasma membranes,^34^ facilitating viral budding^32−37,39^, the clustering mechanism remained unresolved. Both TM and AH domains appear to be important. The M2 AHs utilize the steep dielectric at the lipid–water interface and the water concentration gradients (between the hydrophobic core and lipid headgroup region) to produce membrane undulations^24−32,49,70^ and increase lateral pressure during budding.^36,91^ On the other hand, the TM domain, either in M2TM or M2TM-AH, spans the bilayer and induces the formation of an ordered lipid domain.^34,36,37,49,92^ The increased lipid ordering and generation of the suitable membrane curvature add further strain to the already constricted, phase-separated bud neck, promoting additional constriction and causing membrane scission during *in vivo* budding.^32−37,39^ However, the catenoid-shaped (*K* < 0) influenza budding neck has a diameter of 25.97 ± 11.25 nm,^33^ i.e., much larger than that of spontaneous necks arising due to M2 in SUVs (4.67 nm).^26,70^ [Note: this calculation assumed similar bending rigidity between the SUVs and the membrane neck of a budding virus, possibly underestimating M2’s constricting capacity *in vivo*.] Nevertheless, it has been suggested that the scaffolding-induced constriction and phase segregation alone are insufficient to cause membrane scission in the absence of M2.^30,32−34,39^ Rather, the combination of scaffolding-induced constriction and curvature induced by M2 is sufficient to cause membrane scission.^26,33,34,70^ Therefore, M2 may be necessary to cause scission in preconstricted necks^30^ by reducing the neck diameter by an additional 5 nm by exerting Gaussian curvature of −0.04 nm^–2^^26^ at the midpoint of the neck catenoid.^30,93^

In addition to the native membrane asymmetry, the distribution of cholesterol in the budozone raft-like domain^51−53,68,69^ influences protein organization. There have been several observations that cholesterol impacts M2-driven membrane remodeling. Indeed, there is significant correlation between the AH-cholesterol interactions and both elevated membrane curvature and reduced amantadine binding.^29,94^ Eliminating either component (i.e., AHs or cholesterol) diminishes membrane curvature, thereby affecting the packing of the TM domain and reducing drug accessibility to the pore.^29,94^ Increased bilayer thickness, another well known cholesterol effect, also leads to a more compact homotetramer.^95^ Due to cholesterol’s condensing properties,^96^ the AHs shift away from the membrane in response to cholesterol supplementation. However, cholesterol is not essential for the association of the amphipathic helix with membranes (in LUVs)^97^ nor M2 ion channel function and inhibition by rimantadine,^98^ challenging the assumption of its functional necessity. Another suggested mechanism involving cholesterol is that cholesterol induces a shift in the conformational equilibrium of M2 toward a predominantly α-helical state, promoting clustering^99,100^ and interactions with the helical M1 protein.^34^ In low-cholesterol membranes, the tilted M2TM-AH peptide has a direct effect in membrane remodeling by increasing lipid order, likely via membrane line tension.^54^ High cholesterol levels enhanced the separation of anionic lipid headgroups, and the membrane-parallel insertion of the M2TM-AH protein channel reduced headgroup separation without affecting lipid order.^54^ Lastly, M2, influenced by cholesterol, induces negative membrane curvature, especially in virus-budding lipid raft sites.^33^

In addition to the numerous biophysical and membrane remodeling mechanisms suggested for cholesterol in M2 pathophysiology, it has been shown that cholesterol can directly interact with specific M2TM-AH residues.^27,55−57,65^ ssNMR with labeled cholesterol revealed proximity to residues I39, I42, and F47 of the M2TM-AH channel with a 2:1 stoichiometry for the cholesterol:M2TM-AH complex. Moreover, cholesterol involvement in M2TM-AH clustering has been suggested to be mediated via direct M2TM-AH–cholesterol contacts based on interprotomer distances between M2TM-AH channels using ^19^F ssNMR.^31^ Macroscopically, membrane protein crowding has been known to induce large spontaneous curvature.^101^ However, there was no direct interpretation of the cholesterol-mediated M2 clustering and membrane undulations at the molecular level.

In the present work, we aimed to (a) provide a microscopical description of how cholesterol mediates M2 channel clustering with subsequent effects of the clustering on membrane morphology and (b) provide molecular insight into previous experimental findings. MD simulations can contribute to understanding viral structure, functional dynamics, and processes related to the viral life cycle.^102^ Here, we provide a model for the multimeric formation of M2TM-AH and the membrane curvature induced by M2TM-AH in planar membranes. Using 10 μs repeats of CG MD simulations of M2TM-AH and M2TM in DMPC, POPC, and POPC/POPS without or with cholesterol, we showed that M2TM-AH channels are cluster, as previously suggested^24,28,31,33^, forming multimers through PPIs between AHs in adjacent channels. M2TM lacking the AHs also formed clusters, although to a lesser degree, through the direct attraction between parallel TM helices. The stabilizing PPIs between M2TM-AH protomers consisted of contacts between polar residues at the N-termini of the TM helices and between residues at the C-termini of the AHs in adjacent channels (Figure 3). The multimers formed during the simulation are mainly dimers and trimers (and even pentamers), while the supplementation of cholesterol as a membrane component favors the formation of higher-order multimers (Figures 1 and 2). In POPC/cholesterol and POPC/POPS/cholesterol, all of the clusters have cholesterol molecules bridging paired M2TM-AH channels (Figures 3, 5, 6, and 7). Indeed, our PMF(US) calculations of M2TM-AH channel dimerization in POPC and POPC/cholesterol membranes (Figure 8) showed ΔΔ*G*_POPC→POPC:cholesterol_ = −11.13 kJ/mol (−2.65 kcal/mol), which corresponds to M2TM-AH-M2TM-AH dimers being populated by a factor of 71 compared to monomer species. We therefore propose that cholesterol acts as a molecular glue, making binding between two M2 proteins tighter and increasing the level of multimeric clustering.

In terms of the lipid compositions simulated, we used symmetric planar bilayers composed of DMPC or POPC as reference membrane models to investigate the specific effects of either cholesterol or POPS as an example of an anionic lipid. In the field of M2, and in general, when membrane proteins are reconstituted in proteoliposomes, the experimental data typically use simple lipid compositions due to the practical difficulties in the asymmetric self-assembly of bilayers. Examples include ssNMR structural studies of M2TM in DMPC^16^ (where DMPC matches with M2TM’s hydrophobic length) or M2TM-AH in lipids with longer aliphatic chains (e.g., DOPC/DOPE)^11^ or M2FL in DMPC/DMPG bilayers.^55^ The membrane remodeling effect by M2TM-AH was studied using ssNMR and CG MD simulations in DOPC/DOPE bilayers,^28^ and M2TM-AH clustering affected by cholesterol was studied using ssNMR in POPC/POPG/cholesterol or POPE bilayers.^31^ In other studies, the bending effect in membranes exerted by M2TM-AH and M2TM was investigated in DOPS/DOPE liposomes using small-angle X-ray scattering (SAXS).^26^ Another study reconstituted M2TM-AH in POPC:cholesterol or POPC/POPG/cholesterol liposomes for their measurements using an array of biophysical techniques.^30^ The experimental work done so far with model membranes aims to describe cholesterol effects in viral budding. However, the basis has not been described by using models that enable molecular-level interpretation. Considering these data, we performed our computational experiments using symmetrical lipid mixtures composed of selected key lipid types known to influence curvature and viral budding. Our system selection lies in between models including natural asymmetric lipid membranes and a reductionist *in vitro* biochemical approach, with the aim to interrogate the role of cholesterol.

Nevertheless, alongside our symmetric bilayer compositions (reflective of those used in experimental setups), we have also used a more native-like asymmetric plasma membrane model (see the Methods Section; including different leaflet PC/PE distribution, relatively high cholesterol, and higher levels of glycosphingolipids) and used this to expand our analysis. We postulated that the lipid makeup could influence protein clustering dynamics. We found that M2 channels were still able to cluster in a more heterogeneous plasma membrane mimetic bilayer, creating higher order (>2) oligomers compared to the simple symmetric POPC environment where clustering was limited to dimers in our analysis of 10 μs repeat simulations (see Figure S8). Notably, monosialodihexosylganglioside lipids (GM3) tend to create a tight perimeter around M2 copies, increasing the interprotomer distance. The presence of this lipid annulus complicates the analysis of protein–protein interactions, preventing us from analyzing them in the same way as for the simpler membrane models, requiring different PPI interaction cutoffs to be optimized and ± GM3 control simulations. However, the stability of protein complexes remains evident, as illustrated by the consistent distance between the COMs of M2TM-AH channels (Figure S8c). The clusters in the plasma membrane mimetic bilayer are similar to those formed in the symmetric POPC/cholesterol and POPC/POPS/cholesterol environments (Figure 1). While studies on GM3 as an annular lipid are mostly restricted to MD studies, it has been shown experimentally that GM3 does form clusters in the membrane.^103,104^ It becomes evident that protein–lipid and potentially lipid–lipid interactions can affect clustering outcomes, especially when considering that ganglioside GM3 lipids display an increased affinity for M2 by forming a tight lipid annulus. Physiological GM3 could further augment M2 clustering by dragging M2s into GM3-enriched membrane patches. This phenomenon could be explored in more depth, necessitating an analytical approach that considers the lipid annulus. As this would require extensive work beyond the scope of the present analysis, we reserve this for future work, especially since GM3′s effect on M2 membrane dynamics is entirely unexplored. Concluding, it is essential to recognize the role of lipid composition when exploring protein–lipid–protein interactions. A path for future M2 clustering experiments could be geared toward emulating membrane complexity using both lipid extracts and chemically defined complex bilayers.

We calculated the time-averaged density of bridged cholesterol molecules from 2 × 50 μs CG MD of a trimer of M2TM-AH channels in POPC/cholesterol membrane, and we identified three cholesterol binding sites bridging M2TM-AH channel pairs with distinct residue interactions (Figures 5 and 6). By defining the binding sites using a recently published community analysis method, we identified two sites of high occupancy toward the M2TM-AH N-termini in the top leaflet,^65^ not experimentally observed previously.^55−57^ Importantly, the two top leaflet sites differed in their membrane accessibility, with the site exposed to the convex trimer interface geometry (Figure 5) showing higher occupancy and cholesterol residency compared with the one in the concave interface. Another cholesterol site of high occupancy/high residency (i.e., tight binding and slow exchange with bulk) was identified at the TM and AH joint in the bottom leaflet, which has also been reported by ssNMR.^55−57^ The cholesterol binding site in the bottom leaflet is more stable than those in the top leaflet, potentially explaining why it was detected experimentally whereas the top-leaflet sites were not. The analysis of the per residue residence time showed that in the bottom leaflet cholesterol forms polar interactions with D44, R45, and S50 and has very frequent hydrophobic contacts with L26, I35, L36, H37, I39, L40, I42, I51, and F55. Conversely, the top leaflet binding sites are stabilized through supporting polar interactions with S22 and D24 and frequent van der Waals interactions with P25, A29, I32, and V28. The specific contribution of each site residue was ranked with per-residue residence time (Figure 7).

We report that M2 clustering and M2-induced membrane curvature are favored by cholesterol, in agreement with previous findings.^31−33,36,37,48−50,105^ Thus, PPIs between adjacent M2 channels can be divided into two categories: (A) PPIs involving interacting amino acid residues located at the N- and C- termini and (B) cholesterol-mediated PPIs, referred to as cholesterol bridges, which enable protomers to come into proximity and form larger clusters. The latter mechanism involves a reduction in the free energy of dimerization mediated by cholesterol bridges. Specific cholesterol binding sites attract cholesterol molecules to fill the gap between the hydrophobic cores of two adjacent M2s and promote M2 dimerization.

Upon examination of the effect of lipid-tail length on M2TM and M2TM-AH channel clustering, it became apparent that M2TM channels demonstrated a higher propensity for clustering in DMPC than M2TM-AH channels (Figure 1; Figure S1). On the other hand, in POPC, M2TM-AH channels exhibited higher clustering levels, while the clustering of M2TM channels was significantly diminished. This observation suggests a lipid-sensitive packing effect on M2TM, particularly when AHs are present. As discussed in our previous work, in which we investigated M2TM and M2TM-AH protomers in different lipid environments through AA MD simulations,^65^ both systems exhibited membrane thickness convergence to the same value regardless of the construct. Therefore, M2’s adaptation mechanism to adjust to different membrane thicknesses (i.e., M2TM in POPC or M2TM-AH in DMPC) might involve structural changes that hinder either protein diffusion or aggregation. Consequently, carefully selecting the combination of M2 construct and lipids when studying M2’s clustering mechanism is crucial. Since the choice of the M2 construct plays a crucial role in the clustering mechanism, as shown here and by others,^24,28,31,33^ further investigation is needed, including M2FL in various membrane compositions.

We observed that including the AHs in the M2 construct resulted in greater local deformation in planar lipid bilayers (Figure 2; Figure S10). The clusters in M2TM-AH systems assist valley formation or are spontaneously trafficked to existing membrane valleys, or both, in a synergistic manner. Cholesterol enhances the formation of multimers and valleys. In contrast, in the equivalent M2TM simulations, clusters are located in both the membrane valleys and hills, and there is no obvious correlation between cluster *xy* position and local membrane curvature (Figure S10). We measured the Gaussian curvature (*K*) that M2 channel constructs can generate on the membrane (Figure 9). While the lack of AHs (M2TM) resulted in time-averaged planar membranes, adding AHs on M2 is enough to generate multiple negative Gaussian curvature events with *K* < −0.04 nm^–2^ across the membrane plane. The effect is pronounced with the addition of cholesterol, demonstrating that the steroid can link multiple aspects of M2 physiology by (a) binding to selected high and low exchange rate sites between M2 protomers to create (b) protein–cholesterol–protein bridges that thermodynamically favor dimer populations by 1.5–2 orders of magnitude, which in turn (c) drives channel clustering into conical multimer shapes that exert lateral force on the membrane to induce (d) the necessary negative Gaussian curvature profile to permit spontaneous scission of the catenoid membrane neck and lead to (e) viral buds and scission.

Our analysis makes it difficult to discern whether membrane curvature primarily drives M2 channel clustering or whether M2 channel clustering in the presence of cholesterol bridges can create conical protein formations that force the membrane into a curved shape. In our opinion, these processes are coupled to each other. During the reviewing process of this work, another simulation-based study using MD-based continuum models focusing on membrane curvature sensing by M2 protomers was published by the Grabe group.^106^ While the authors did not explicitly model PPIs or protein–lipid interactions as we have done here, they uncovered that in membranes implicitly modeled to contain 30–50% cholesterol, C4-symmetric (PDB: 2L0J(107)), C2-symmetric (PDB: 2N70(108)) M2TM-AH protomers and an MD-generated lipid-relaxed C2-symmetric atomistic model all preferentially sorted to inward-budding, spherical caps (similar to what we refer to as “valleys”) of concave curvature. Notably, only their C2-symmetric MD model could be moderately stabilized in saddle-shaped membranes (where *K* < 0; having matched C2-symmetry, with κ_1_ = κ_2_) by 4 kT, recognizing that C4-symmetry-breaking, is likely essential to localize M2 to the catenoid budding neck. This data further supports our negative Gaussian curvature generation findings in systems with M2 clustering (e.g., C2-symmetric dimers, trimers) and cholesterol. Grabe *et al*. also modified the continuum model’s parameters to match a cholesterol-free bilayer and found the C2-symmetric model’s preference for saddle-shaped geometries was largely abolished, complementing the central role of cholesterol we report here. While both studies recognize cholesterol’s role in membrane curvature and M2 stabilization, our work uses explicit PPI and protein–lipid modeling (limited to planar membranes) and focuses on how cholesterol acts as a “molecular glue” that stabilizes M2 multimers by filling gaps between M2 protomers, significantly enhancing the clustering process, a key step for effective curvature generation. We further fill the mechanistic gap by providing detailed insights into the cholesterol binding sites, which they did not observe, likely owing to the limited sampling in their initial all-atom simulations and the lack of modeling of multiple protomers. The linear M2 clustering presented here could further stabilize C2-symmetric structures in saddle-shaped membranes by more than 4 kT using the Grabe group’s continuum method. This would be an interesting step forward in the mechanistic understanding of influenza A viral budding. Indeed, C2-symmetric protomers of WT and S31N M2 have been resolved in structural NMR studies.^108,109^

Other studies using CG MD simulations and generic lipid bilayer models^110,111^ suggested that once a minimal local bending by a protein is realized, the effect robustly drives protein cluster formation and subsequent transformation into vesicles with radii that correlate with the local curvature imprint.^112^ In these simulations, when small proteins were placed in a simulated lipid environment similar in scale to lipid microdomains, vesicular structures formed spontaneously despite a lack of interprotein interactions. Instead, the vesicularization was induced by the local hydrophilic attraction between the proteins and their immediate lipid environment. These local attractions led to aggregation and local membrane curvature, which summed over the membrane to induce vesicle formation. For example, it was found that the matrix (M) protein of the vesicular stomatitis virus alone was able to impose the correct budding curvature on the membrane using confocal microscopy in giant unilamellar vesicles (GUVs) consisting of pure DOPC or from a mixture of DOPC /DOPS (9:1).^113^ Additionally, when virus-capsid-sized particles were placed in the lipid environment, these also induced spontaneous vesicle formation.

Notably, by the time this manuscript was in the revision process, cholesterol parameters for Martini 3 had been published.^114^ The utilization of the MARTINI force field in simulation studies has yielded valuable insights into the association of membrane proteins^77,79,80,115−118^ and protein–lipid interactions.^59,119,120^ It has been reported that the MARTINI 2.2 model (used in this study) has certain limitations,^121,122^ notably smoothing free energy landscapes, which may lead to accelerated oligomerization kinetics. MARTINI 2.2 utilizes a shifted function with a cutoff at 1.1 nm and implicit screening to depict electrostatic interactions, instead of relying on the particle mesh Ewald approximation. This, in turn, may lead to an underestimation of long-range electrostatic interactions between proteins. Consequently, interactions involving charged lipids and the protein through electrostatic forces could be overestimated. Nevertheless, clustering of the M2 channels was also previously observed in CG MD simulations^24^ using the BPB force field.^123,124^ Additionally, it is essential to emphasize that the key lipid of interest in this study is neutral cholesterol and its interactions occur within relatively short distances (less than 1.1 nm). A just-published work reported the reliability of cholesterol Martini 2.2 parameters based on the agreement between simulations and experimental results.^87^ Importantly, our CG MD simulation results agree with the experimental evidence suggesting cholesterol molecules between M2TM-AH channels.^31^ Nevertheless, subsequent investigations into M2 oligomerization using MARTINI 3^125^ are poised to be intriguing, especially if nonplanar membrane geometries imposed with boundary conditions are considered in parallel, as demonstrated recently by Grabe *et al*.^106^ Additionally, after the submission of this manuscript, Kim *et al*. demonstrated that for very large membrane systems (100 nm × 100 nm × 80 nm, > 30,000 lipids and >6.5 million waters) the default neighbor list update step and interaction cutoff, *r*_c_, used in MARTINI 2.2 simulations need to be refined to avoid artificial curving of a lipid membrane.^126^ Our simulated systems are smaller (40 nm × 40 nm × 10 nm, < 5,000 lipids, < 80,000 waters) and we did not observe excessive artificial curving in protein-free control POPC bilayers (Figure 9; Figure S6).

## Conclusions

Cholesterol is the molecular ingredient commonly associated with protein lateral diffusion and mobility.^53,127,128^ It is possible that the lateral diffusion rates of M2TM-AH protomers and other associated viroporins through the membrane to engage in clustering are affected in the presence of cholesterol, thereby allowing rapid translocation of the channels across the *xy* plane. Using CG MD simulations, we found that M2 channel clustering and membrane curvature are favored by cholesterol, in agreement with previous findings.^31−33,36,37,48−50,105^ Higher-order multimers promote membrane curvature in the cholesterol-dense membrane during viral budding. Cholesterol concentration can play a dual role in M2 mesoscale dynamics by balancing (a) the viroporin’s self-dimerization affinity and (b) the probability of multimerization. It is likely the latter proceeds via a membrane fluidity modulation mechanism, for which cholesterol is known, but this remains to be tested. The simulations presented here inform our understanding of the mesoscale organization of influenza A M2 channels in host cell membranes during virus budding. These findings may have implications for developing novel medications for influenza virus infection via inhibition of cluster formation and will trigger related research for other viruses.^47^ It has been suggested that membrane remodeling can contribute to virus budding effected by the matrix protein 1 (M1) of influenza C,^129^ the nonstructural protein 1, and the Ebola Virus Matrix Protein VP40.^130^ The SARS-CoV-2 homopentameric protein E forms clusters^131^ and also causes membrane bending.^132,133^ It has been suggested for HIV that cholesterol increases the clustering of viroporin gp41 channels wherein cholesterol bridges multiple gp41 trimers, increasing proximity and facilitating virus-cell fusion and virus budding.^134,135^ Overall, the general consensus appears to centrally involve cholesterol in membrane remodeling and viroporin clustering, correlating well with the high concentration of this lipid in membrane rafts from where these viruses initiate budding.

## Methods

### System Setup for CG MD Simulations

We performed the simulations shown in Table S1. For the M2TM CG MD simulations, we used the final snapshot obtained from a 200 ns-AA MD simulation starting from the experimental structure of M2TM with PDB ID 2KQT as the starting structure.^16^ We converted the AA model of M2TM to a CG model using the *martinize.py* script (version 2.4), available on the MARTINI coarse grain force field website (http://md.chem.rug.nl/index.php/tools2/proteins-and-bilayers). We then applied the MARTINI2.2 force field^64,72,73^ and the ElNeDyn elastic network model.^118^ The CG model of the M2TM channel was embedded into a membrane bilayer of the desired lipid composition, which was solvated using the standard MARTINI water model^64,72,73^ and neutralized to the physiological 0.15 M NaCl concentration using the *insane.py* script^136^ available on the MARTINI Web site. This system of M2TM channel in a simulation box dimension with 10 × 10 × 10 nm was minimized, equilibrated, and simulated for 1 μs. We used the final snapshot of the M2TM channel system to assemble a larger system of 4 × 4 copies of the M2TM channel, using the GROMACS^137,138^ tool genconf, consisting of 16 proteins and ∼5000 lipids. This system of 16 M2TM channels was subjected to CG MD simulations for 10 μs.

For the 10 μs CG MD simulations of M2TM-AH systems, we also started from the final snapshot obtained from a 200 ns MD simulation of M2TM-AH embedded in a hydrated lipid bilayer consisting of DMPC or POPC or POPC/POPS, ± 20% cholesterol, starting from the experimental structure of M2TM-AH with PDB ID 2L0J.^107^

We performed GG MD simulations, testing M2TM-AH channel clustering in a plasma membrane mimetic model. The extracellular leaflet of the plasma mimetic membrane is enriched in phosphatidylcholine (PC) lipids, while the intracellular leaflet is enriched in phosphatidylethanolamine (PE) lipids and phosphatidylserine (PS) lipids. Thus, the upper leaflet consisted of 4:4:1:1:3:2:5 POPC:DOPC:DOPE:POPE:SPH:GM3:cholesterol while the lower leaflet was 1:1:4:4:2:1:2:5 POPC:DOPC:POPE:DOPE:POPS:DOPS:PIP2:cholesterol.

To perform the 50 μs CG MD simulation starting from a trimer of M2TM-AH channels in POPC/cholesterol, we used a trimer of M2TM-AH channels with cholesterol molecules bridging the protomers from the last snapshot of the 10 μs CG MD simulation of M2TM-AH in POPC/cholesterol; in this trimer, each cholesterol molecule was between the protomers in a distance of 9 Å between the COM of cholesterol and the COM of each M2 protein. We extracted this trimer from the trajectory mentioned above using VMD.^139^ The trimer of M2TM-AH channels was embedded into a POPC/cholesterol 20% membrane bilayer, solvated using the standard MARTINI water model^64,72,73^ and neutralized to a 0.15 M NaCl concentration (Table S1), using the *insane.py* script. The resulting system had simulation box dimensions of 12 × 14 × 10 nm and was minimized, equilibrated, and subjected to CG MD simulations for 50 μs repeats.

### CG MD Simulation Parameters

We performed all simulations using GROMACS 5.1.2 (www.gromacs.org)^137,138^ and the MARTINI 2.2 force field.^64,72,73^ In all CG MD simulations, we applied periodic boundary conditions and a time step of 20 fs. The temperature was maintained at 320 K using the Berendsen thermostat^141^ and the pressure was maintained at 1 bar using the Berendsen barostat,^141^ except from the simulation of the trimer of M2TM-AH channels, where a velocity rescale thermostat^71^ and Parinello–Rahman barostat^142^ were used. A coupling constant of 1 ps was used for temperature coupling, and a coupling constant of 10 ps was used for pressure coupling. The electrostatic interactions were calculated using a coulomb-type potential, and for van der Waals interactions, a switching function from 0.0 to 1.1 nm and a cutoff distance at 1.1 nm were used. The LINCS algorithm was used.^143^ All systems were subjected to CG MD simulations for 10 μs repeats except the trimer of the M2TM-AH channels system, which was simulated for 50 μs repeats.

### Backmapping and AA MD Simulation Parameters

We carried out backmapping of the CG MD simulation snapshots to AA models in CHARMM36m force field^82^ using CG2AT2.^144^

For selected systems (see Table S1), AA MD simulations were performed using the CHARMM36m.^82^ Initial structures were obtained by transferring the equilibrated structures from the CG MD simulations to atomistic resolution with approximately 168,000 atoms. The temperature was kept at 320 K via the v-rescale algorithm.^71^ Semi-isotropic pressure coupling to 1 bar was applied using the Parrinello–Rahman algorithm^145,146^ with a compressibility of 4.5 × 10^–5^ bar^–1^, van der Waals forces were smoothly switched to zero (between 1.0 and 1.2 nm), and the electrostatic interactions were treated with the PME method.^147^ The time step was 2 fs. The all-atom systems were studied at a salt concentration of 0.150 M NaCl. The systems were subjected to AA MD simulations for 500 ns independent triplicate repeats, starting from randomized velocities.

### Cholesterol Binding Site Identification and Analysis

The 50 μs trajectories of the isolated trimer of M2TM-AH channels in POPC/cholesterol and POPC/POPS/cholesterol were analyzed with the PyLipID^74^ python library. In PyLipID, the cholesterol-interaction cutoffs specified were 0.475–0.75 nm. Cholesterol occupancy and density visualization were carried out with VMD 1.9.4.^139^

We selected a representative trimer frame that captured the average cholesterol profile (*t* = 8.116 μs for POPC/cholesterol) for further analysis. This was done by binning the calculated cholesterol occupancy to an isosurface of isovalue 0.08 and superimposing the 50 μs trajectory (VMD 1.9.4^139^ VolMap tool), frame-by-frame.

### Protein–Protein, Protein–Lipid Interactions, and Clustering Analyses

PPIs and protein–lipid interactions were identified using in-house clustering scripts using the NumPy^148^ and MDAnalysis^140^ modules. Residues of neighboring proteins were considered to interact when residue centroids were within 0.8 nm of one another. Similarly, in-house scripts using the NumPy, MDAnalysis, and NetworkX Python libraries were used for the clustering analysis. In the clustering scripts, M2 channels were considered to interact when the centers of mass of the whole protein were within 3.9 nm of each other (for M2TM-AH) and 2.7 nm of each other (for M2TM). Graphs were plotted using xmgrace, Gnuplot 4.6 (www.gnuplot.info), and Matplotlib,^149^ and for molecular visualization, VMD^139^ was used. A cutoff distance of 0.6 nm was employed for protein–lipid contact analysis based on radial distribution functions for CG lipid molecules.^150^ The 2D density map was computed by using the VolMap VMD plugin tool. The occupancy density calculations were performed by applying a grid over the simulation box with 1 Å resolution and were averaged across all frames and normalized. The rotational and translational motions of the protein were alleviated by fitting the protein backbone using the trjconv -fit option in GROMACS and the VMD RMSD alignment tool. Scripts used to analyze PPIs can be found at https://github.com/annaduncan/clustering_prot, and scripts to analyze protein–lipid interactions at https://github.com/annaduncan/Kir_scripts/blob/master/lipid_prot_interaction_frequencies_v5.py

### Umbrella Sampling PMF Calculations

We performed all protein–protein PMF calculations with GROMACS^137,138^ 2020.3. The systems consisting of dimers of M2TM-AH channels were prepared for PMF(US) calculations as follows. A representative frame from the 50 μs CG MD simulations trajectory (*t* = 8.116 μs) of the trimer of M2TM-AH channels was selected with all interfacial cholesterol sites occupied. The trimer of the M2TM-AH channels consists of two adjacent topological dimers. One of the two topological dimers in POPC/cholesterol was selected for subsequent PMF calculations by removing the coordinates for the third M2TM-AH protomer. After removal of an M2TM-AH protomer, the truncated system was supplemented with ions to reach 150 mM NaCl and charge neutrality. The M2TM-AH_dimer_ system was subjected to two rounds of steepest descents minimization followed by bilayer self-assembly after 1 μs equilibration to fill the membrane gap caused by the removed protomer. The self-assembly step was done in NPT with a conservative 10 fs time step and with the remaining parameters as in the CG MD simulations section above. The equilibrated dimer of M2TM-AH channels in the POPC/cholesterol system was used to prepare an identical system without cholesterol. 113 cholesterol coordinates were deleted, and the dimer M2TM-AH in POPC was equilibrated by another round of bilayer self-assembly. Position restraints were applied on the protein backbone in the dimer of M2TM-AH channels using MARTINI beads (*k*_*xy* plane_ = 1000 kJ/mol/nm^2^), thus yielding starting channel dimer coordinates identical to the + cholesterol system.

Having carefully prepared comparable dimers of M2TM-AH channels in POPC/cholesterol and POPC bilayers with or without cholesterol between the M2TM-AH protomers, the starting configurations were used in steered MD to drive dissociation. The dissociation of the dimer of M2TM-AH channels into protomers was driven by linearly increasing the *x–y* plane distance between the protomers’ COM at a constant rate of 0.0001 nm per ps and with a force constant 1000 kJ/mol/nm^2^. The COM distance collective variable ranged from 30.0 to 56.0 Å, and system snapshots were selected every 0.5 Å, for a total of 95 windows per system. The PMF curves plateaued to bulk values within the selected range. For the US windows, the force constant was 10 kJ/mol/nm^2^ for 3–4.5 Å and 1000 kJ/mol/nm^2^ for 4.5–5.6 Å. Orientational restraints were applied to the protomers to prohibit undesired rotation about the M2TM-AH *z*-axis, which would increase the time required for convergence. This was achieved by applying a rotational potential to the particles of each protein. This is equivalent to setting the rotation matrix Ω(*t*) to 1 for particle entries. The potential chosen does not influence the positional umbrella potential since it has been proved^151^ that radial forces and forces parallel to the rotation axis are eliminated by using the rotational potential form according to
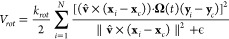
where **v̂** is a unit vector parallel to the rotation axis; **x**_*i*_ and **y**_*i*_ are the current and reference positions of the *i*^th^ MARTINI particle, respectively; **x**_*c*_ and **y**_*c*_ are the current and reference positions of the center of mass of the *N* MARTINI particles in each protein, respectively; **Ω**(*t*) is a rotation matrix, which describes the motion of the potential; *k*_*rot*_ is the force constant for the rotational potential, and ϵ is a small constant required to avoid a singularity at the axis of rotation.^83^

This method has been previously used to calculate NanC dimerization free energies.^83^ The rotational potential is applied via the enforced rotation code in GROMACS (rot_type0 = rm2-pf in the mdp file), which applies an appropriate translation force to each restrained particle to avoid rotational torques. *k*_*rot*_ = 1000 kJ/mol/nm^2^ and ϵ = 0.01 nm^–2^ were selected as previously reported to be appropriate for a membrane protein system.^83^ Each US window was equilibrated for 200 ns in NPT followed by 4.8 μs of production time, for a total of 518.4 μs window sampling time. Within this time, the PMFs obtained converged. To reconstruct the window-mean forces into a PMF curve, gmx wham^84,85^ was used with 200 bootstrap rounds, 200 bins, and a tolerance of 1 × 10^–06^. The average profile and error bars extracted from gmx wham were plotted in Python 3 with the matplotlib library.

### Gaussian Curvature (*K*) Measurements

Gaussian membrane curvature (*K*) was calculated using in-house Python scripts, using NumPy,^148^ SciPy,^152^ and the MDAnalysis^140^ module as implemented in the MembraneCurvature package version 0.0.2.^153^ The phospholipid phosphate beads (PO4) were selected as reference points for grid generation, and the average Gaussian curvature was calculated over two independent repeat simulations and over (a) the last 100 ns to capture the fluctuation range better or (b) the last 2 μs to provide better curvature statistics.

## Data Availability

Information about simulation systems setup, software, and scripts used are provided in the Methods section. The simulation input coordinates (gro, pdb files) and parameter files (mdp, itp files) of the CG MD simulations described in Table S1 are available via https://github.com/dimkol94/M2-cholesterol-clustering.

## Supporting Information Available

Detailed descriptions of the simulated systems (Table S1); extracted cholesterol kinetic parameters (Table S2); descriptive statistics of Gaussian curvatures (Table S3); formation of clusters of M2 channels over the duration of 10 μs-CG MD simulations (Figure S1); angle between M2TM or M2TM-AH bundle axis and the membrane normal over the simulation time (Figure S2); protein–lipid interaction plots associated with Figure 4 (Figure S3); PMF profile and histogram analysis for the dissociation of dimer of M2TM-AH channels in POPC (Figure S4); PMF profile and histogram analysis for the dissociation of dimer of M2TM-AH channels in POPC/cholesterol with three cholesterols occupying the interfacial binding sites (Figure S5); scatter plot of Gaussian curvature values (Figure S6); absolute values of the mean Gaussian curvature (|*K*|) from different systems (protein-free, M2TM, M2TM-AH) as a function of trajectory time (Figure S7); comparison of M2AH clustering in POPC versus a plasma membrane mimetic model (incl. GM3 lipids) (Figure S8); cluster stability analysis of M2TM-AH dimers in POPC and POPC:cholesterol (4:1) from atomistic MD simulations with CHARMM36 (Figure S9); representative snapshots of local membrane deformation and formation of M2 channel clusters from CG MD (Figure S10); supplementary heatmaps of Gaussian curvature (*K*) induced by M2 constructs (±AHs) and cholesterol from 10 μs CG MD of 16 copies of the M2 constructs and protein-free control bilayers averaged over different time-scales (final 100 ns versus final 2 μs) (Figures S11–S13)(PDF)

## Abbreviations

AH: amphipathic helix
ER: endoplasmic reticulum
ESCRT: endosomal sorting complexes required for transport
CG MD: coarse-grained molecular dynamics
DOPC: 1,2-dioleoyl-*sn*-glycero-3-phosphocholine
DOPE: 1,2-dioleoyl-*sn*-glycero-3-phosphoethanolamine
HCV: hepatitis C virus
HIV: human immunodeficiency virus
M2 protein: matrix-2 protein
MD: molecular dynamics
M2TM: transmembrane domain of M2 protein
POPC: 1-palmitoyl-2-oleoyl-*sn*-glycero-3-phosphocholine
POPE: 1,2-dipalmitoyl-*sn*-glycero-3-phosphoethanolamine
POPS: 1-palmitoyl-2-oleoyl-*sn*-glycero-3-phosphoserine
PMF: potential of mean force
PPI: protein–protein interaction
PV: poliovirus
SARS CoV: severe acute respiratory syndrome coronavirus
US: umbrella sampling
vpu: viral protein U
WHAM: Weighted Histogram Analysis Method
